# Circulating inflammatory cytokines and hypertensive disorders of pregnancy: a two-sample Mendelian randomization study

**DOI:** 10.3389/fimmu.2023.1297929

**Published:** 2023-11-16

**Authors:** Siqi Guan, Xiaoxu Bai, Jincheng Ding, Rujin Zhuang

**Affiliations:** Department of Obstetrics and Gynecology, The Second Affiliated Hospital of Harbin Medical University, Harbin, China

**Keywords:** hypertensive disorders of pregnancy, pre-eclampsia, inflammatory cytokine, Mendelian randomization, genome-wide association study

## Abstract

**Background:**

Hypertensive disorders of pregnancy (HDP) pose a significant risk to maternal and fetal well-being; however, the etiology and pathogenesis of HDP remain ambiguous. It is now widely acknowledged that inflammatory response and the immune system are closely related to HDP. Previous research has identified several inflammatory cytokines are associated with HDP. This study applied Mendelian randomization (MR) analysis to further assess causality.

**Methods:**

Patients with HDP who participated in the MR analysis presented with four types of HDP: pre-eclampsia or eclampsia (PE); gestational hypertension (GH); pre-existing hypertension complicating pregnancy, childbirth and the puerperium (EH); and pre-eclampsia or poor fetal growth (PF). A two-sample MR analysis was used to analyze the data in the study. The causal relationship between exposure and outcome was analyzed with inverse variance weighting (IVW), MR Egger, weighted median, weighted mode, and simple mode methods, where IVW was the primary method employed.

**Results:**

Our MR analysis demonstrated a reliable causative effect of Interleukin-9 (IL-9) and macrophage migration inhibitory factor (MIF) on reducing HDP risk, while macrophage inflammatory protein 1-beta (MIP1b), Interleukin-13 (IL-13), and Interleukin-16 (IL-16) were associated with promoting HDP risk.

**Conclusions:**

This study demonstrated that IL-9, MIF, MIP1b, IL-13, and IL-16 may be cytokines associated with the etiology of HDP, and that a number of inflammatory cytokines are probably involved in the progression of HDP. Additionally, our study revealed that these inflammatory cytokines have causal associations with HDP and may likely be potential therapeutic targets for HDP.

## 1 Introduction

Hypertensive disorders of pregnancy (HDP)—including pre-eclampsia/eclampsia, gestational hypertension, chronic hypertension, and pre-eclampsia superimposed on chronic hypertension (1)—is ranked as the second leading cause of maternal death globally, after maternal hemorrhage (2). Apart from causing harm to the mother, HDP is also associated with adverse fetal outcomes, such as preterm labor and fetal growth restriction (3). The exact pathogenesis of HDP is unclear, and current studies have found that endothelial dysfunction, insufficient trophoblastic invasion, abnormal angiogenesis, and aberrant uterine artery remodeling are considered to be key elements in its development (4–6). Among women in the gestational state, inappropriate immune activation and subsequent inflammation may result in maternal vasculopathy or placental dysfunction, thereby contributing to HDP (7).. Previous studies have revealed that certain inflammatory cytokines are strongly correlated with HDP, such as macrophage inflammatory protein 1-beta (MIP1b) and interleukin-18 (IL-18) (8, 9). However, the causal link of various cytokines and HDP is difficult to establish through observational studies because of interference from confounding variables and reverse causations (10).

Mendelian randomization (MR) is an instrumental variable (IV) analysis which utilizes genetic variation as the IV to assess the causality for exposure and outcome (11). The advantages of MR analysis are as follows: first, genetic variations are distributed at random during meiosis and, thus, the likelihood of correlation with environmental confounders is rare (12); second, genotype distribution precedes acquired exposure in time and the two are not impacted by reverse causality; third, exposure-related genetic variation generally accompanies individuals from birth to adulthood, thereby avoiding attenuation due to error in causal inference (regression dilution bias) (13). Additionally, Mendel’s laws of inheritance are universal in the population, thereby avoiding the problem of representativeness in randomized controlled experiments. A two-sample MR analysis enables assessment of the relation between exposure and outcome in two independent populations, thereby expanding the scope of application and improving validity. MR analysis was performed on the basis of the following three hypotheses related to IVs. The first hypothesis is that the selected IVs are genetic variants highly correlated to the exposure. The second hypothesis is that IVs have no association with confounding factors between exposure and outcome. The third hypotheses is that IVs make no difference to outcome directly and merely affect outcome through exposure (14).

The aim of this study is to investigate the causal association between HDP and 41 inflammatory cytokines by using MR analysis and to identify inflammatory cytokines that can serve as potential biomarkers to facilitate early detection and treatment strategies for HDP.

## 2 Materials and methods

### 2.1 Study design

**Figure 1:**
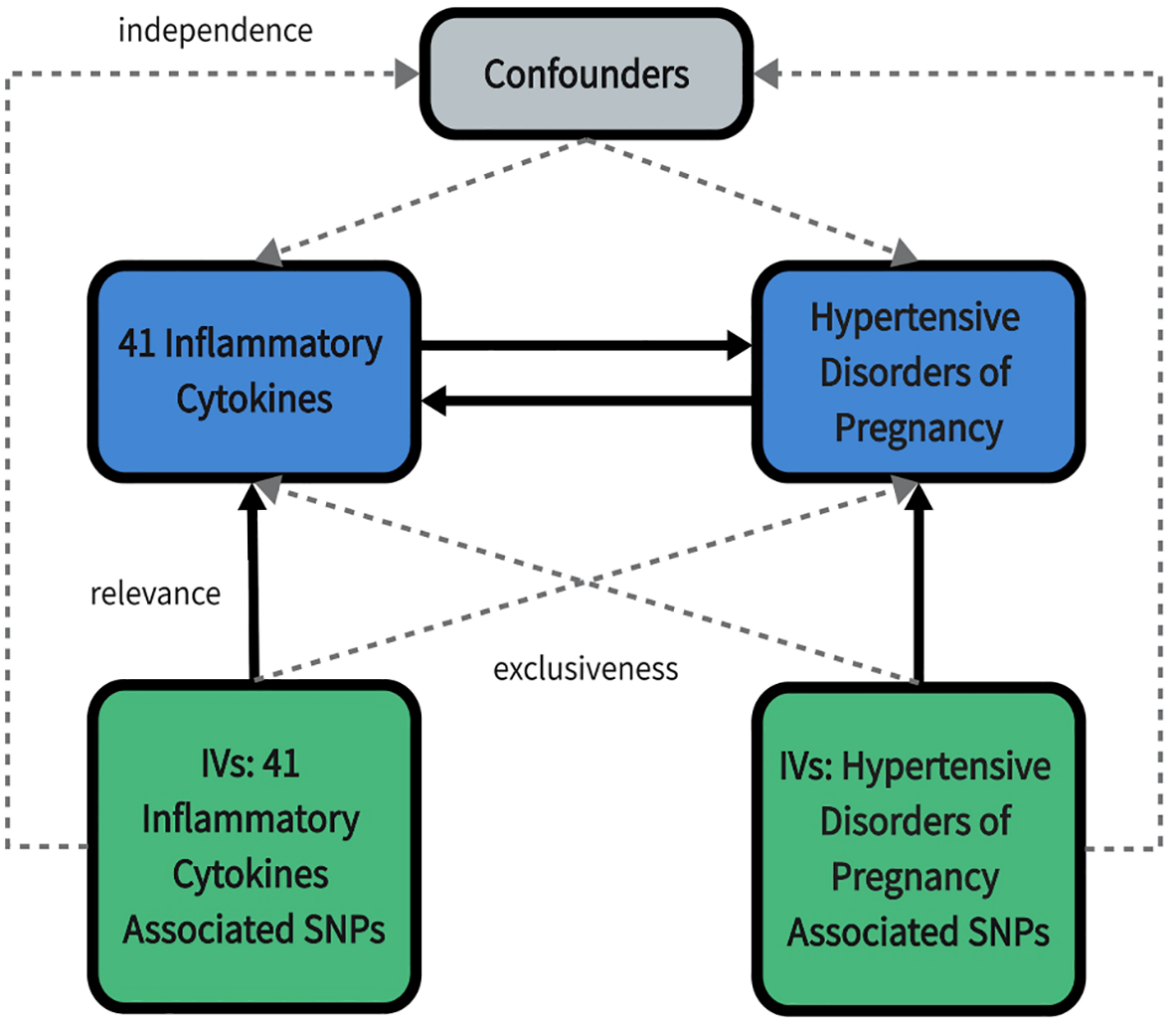
An overall design of the present study. IV, Instrumental variable; SNP, Single nucleotide polymorphism.

We used published summarized genome-wide association studies (GWAS) data for 41 circulating inflammatory cytokines and 4 types of HDP. In our study, a two-sample MR analysis was carried out in order to investigate bidirectional causality between inflammatory cytokines and HDP. This MR study was in accordance with the guidelines of Strengthening the Reporting of Observational Studies in Epidemiology using Mendelian Randomization (STROBE-MR) (15). The study design overview is presented in **Figure 1**.

### 2.2 Data sources

Data on inflammatory cytokines was taken from a GWAS meta-analysis that provided genomic variants in 8293 Finnish individuals (16). This study was conducted in three cohorts including The Cardiovascular Risk in Young Finns Study (YFS) and FINRISK surveys of the 1997 and 2002, which have already been completed. The average age of the participants in the YFS study was 37 years and that in the FINRISK survey was 60 years. The GWAS data of inflammatory cytokines used in this MR analysis were after adjusting for age, sex, and body-mass index (BMI). HDP-summarized GWAS data were retrieved from FinnGen database (https://www.finngen.fi/en) R9 version. According to the definition of HDP we included two cohorts of gestational hypertension; and pre-eclampsia/eclampsia. However, since the GWAS data for chronic hypertension were not available and the pre-eclampsia superimposed on chronic hypertension cohort had only 154 case samples, these two categories under the HDP definition were unable to be included in this MR study. In addition, we found two cohorts with larger sample sizes, pre-existing hypertension complicating pregnancy, childbirth and the puerperium and pre-eclampsia or poor fetal growth, which were thus included in the study. Finally, we selected four cohorts for inclusion in this MR analysis, including pre-eclampsia or eclampsia (PE); gestational hypertension (GH); pre-existing hypertension complicating pregnancy, childbirth and the puerperium (EH); and pre-eclampsia or poor fetal growth (PF). No overlap existed between the exposure and outcome groups in the population. **Table S1** lists details of the four types of HDP.

### 2.3 Instrumental variable selection

First, we applied a genome-wide significance threshold of p < 5 × 10^-8^ so as to filter out single nucleotide polymorphisms (SNP) that were closely associated with HDP and inflammatory cytokines. A few inflammatory cytokines were found under this criterion that did not have a sufficient number of SNPs to perform MR analysis; thus, a higher cutoff value (p < 5 × 10^-6^) was selected. Second, when inflammatory cytokines were used as exposure, we clumped SNPs (kb = 10000, r^2^ = 0.01), which implies that SNPs with r^2^ > 0.01 in the 10000 kb range with the most significant SNPs were eliminated, thereby avoiding linkage disequilibrium (LD). Whereas for HDP as exposure, the number of SNPs was sufficient and, hence, a higher criterion for eliminating LD was set—kb = 10000, r^2 ^= 0.001. As we could not determine whether the palindromic SNPs were oriented in a consistent direction between exposure and outcome, these SNPs were not involved in MR analysis. Third, the R^2^ value was calculated for each SNP, which indicates the extent of exposure explained by IVs; moreover, to avoid weak instrumental bias, we evaluated the strength of the IV correlations by calculating the F-statistic and selected SNPs with F-statistic >10 for inclusion in this MR analysis. Finally, whenever unavailable SNPs are found, we use the LDlink Website (https://ldlink.nci.nih.gov/) to search for proxy SNPs (r^2^ > 0.8) to replace them.

### 2.4 Statistical analysis

MR analysis is conducted utilizing the “TwoSampleMR” R package (Version 0.5.6) of the RStudio (version 4.2.3), which includes five MR analysis methods. Of these five methods, inverse variance weighting (IVW) was employed as the main method, with MR Egger regression, weighted median, simple mode, and weighted mode as complementary methods. The IVW method, which ignores the presence of the intercept term in the regression and takes the inverse of the result variance (quadratic of se) as the weight for the fit, assumes that all of the SNPs turned out to be valid instrumental variables and are completely independent of each other (17). MR Eggar regression accounts for the existence of an intercept term. It assumes that the instrument-exposure and instrument-outcome associations are independent—that is, the instrument strength independent of the assumption of direct effect (18). The weighted median method requires a minimum of 50% of the IVs to be valid to obtain a robust estimate; this method tolerates more invalid IVs (19). Further, inflammatory cytokines that were ultimately identified as statistically significant were subject to the following conditions: The IVW results revealed that the 95% confidence intervals (CI) did not cross over with 1 (or 0), p < 0.05, and met the consistent trend of inflammatory cytokine effects derived from the five MR analyses.

We employed three sensitivity analysis methods to assess the sensitivity of MR results, including the heterogeneity test, pleiotropy test, and leave-one-out sensitivity test. Cochran’s Q test and Rucker’s Q test were used to detect the heterogeneity, and p > 0.05 was regarded as no heterogeneity. The intercept of the MR Eggar analysis results were used for testing the horizontal pleiotropy, with p > 0.05 considered as no pleiotropy (20). Additionally, the MR PRESSO method is capable of outlier identification and horizontal pleiotropy detection simultaneously (21). When applying MR PRESSO analysis, the NbDistribution parameter refers to the number of simulations calculated, and we set this parameter to 1000. Leave-one-out sensitivity was used to investigate whether the causal association was influenced by an SNP between exposure and outcome (22). Further, this study was not pre-registered on any platform.

## 3 Results

**Figure 2:**
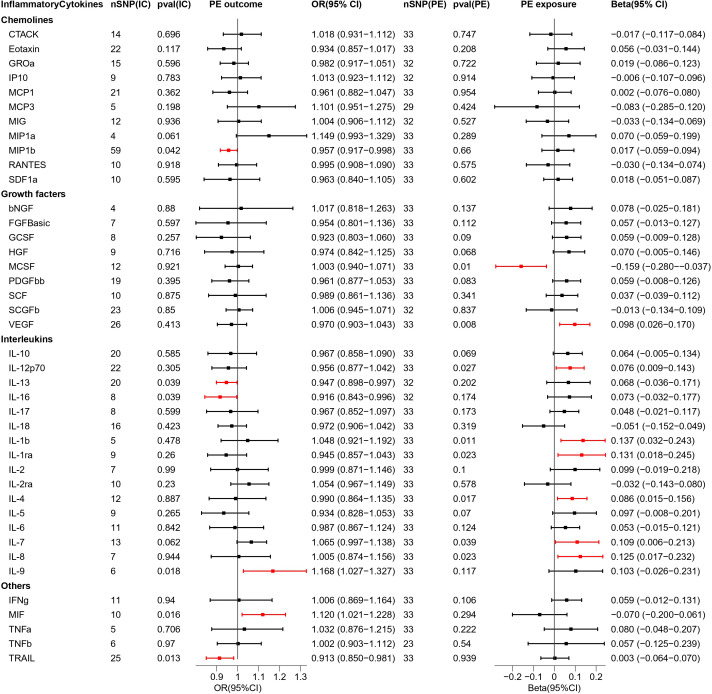
Causal estimations between 41 inflammatory cytokines and PE. The odds ratio (OR) was estimated using the random effect IVW method. The horizontal bars represent 95% confidence intervals (CI). SNP, Single nucleotide polymorphism; IC, Inflammatory Cytokine; pval, P-value; PE, Pre-eclampsia or eclampsia; OR, Odds ratio; CI, Confidence interval; CTACK, Cutaneous T cell-attracting chemokine; bNGF, Basic-nerve growth factor; FGFBasic, Basic fibroblast growth factor; GCSF, Granulocyte colony-stimulating factor; GROa, Gastroesophageal resuscitative occlusion of the aorta; HGF, Hepatocyte growth factor; IFNg, Interferon-gamma; IL-10, Interleukin-10; IL-12p70, Interleukin-12p70; IL-13, Interleukin-13; IL-16, Interleukin-16; IL-17, Interleukin-17; IL-18, Interleukin-18; IL-1b, Interleukin-1 beta; IL-1ra, Interleukin-1 receptor antagonist ; IL-2, Interleukin-2; IL-2ra, Interleukin-2 receptor antagonist; IL-4, Interleukin-4; IL-5, Interleukin-5; IL-6, Interleukin-6; IL-7, Interleukin-7; IL-8, Interleukin-8; IL-9, Interleukin-9; IP10, Interferon gamma-induced protein 10; MCP1, Monocyte chemotactic protein 1; MCP3, Monocyte chemotactic protein 3; MCSF, Macrophage colony stimulating factor; MIF, Macrophage migration inhibitory factor; MIG, Monokine induced by interferon (IFN)-gamma; MIP1a, Macrophage inflammatory protein 1-alpha; MIP1b, Macrophage inflammatory protein 1-beta; PDGFbb, Platelet derived growth factor bb; RANTES, Regulated upon activation normal T cell expressed and presumably secreted; SCF, Stem cell factor; SCGFb, Stem cell growth factor-beta; SDF1a, Stromal cell-derived factor-1alpha; TNFa, Tumor necrosis factor-alpha; TNFb, Tumor necrosis factor-beta; TRAIL, Tumor necrosis factor-related apoptosis-inducing ligand; VEGF, Vascular endothelial growth factor.

**Figure 3:**
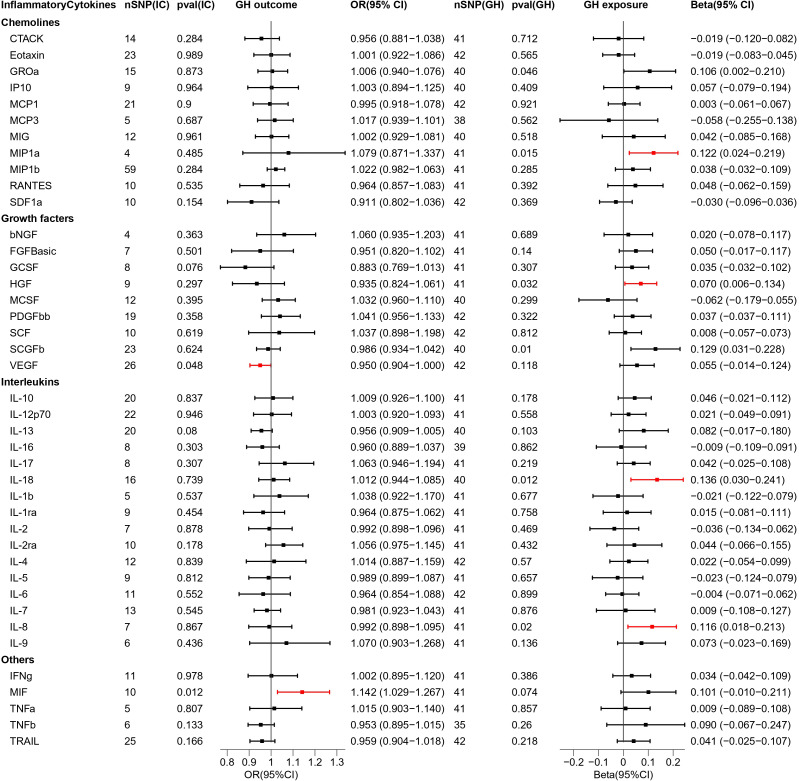
Causal estimations between 41 inflammatory cytokines and GH. The odds ratio (OR) was estimated using the random effect IVW method. The horizontal bars represent 95% confidence intervals (CI). SNP, Single nucleotide polymorphism; IC, Inflammatory Cytokine; pval, P-value; GH, Gestational hypertension; OR, Odds ratio; CI, Confidence interval; CTACK, Cutaneous T cell-attracting chemokine; bNGF, Basic-nerve growth factor; FGFBasic, Basic fibroblast growth factor; GCSF, Granulocyte colony-stimulating factor; GROa, Gastroesophageal resuscitative occlusion of the aorta; HGF, Hepatocyte growth factor; IFNg, Interferon-gamma; IL-10, Interleukin-10; IL-12p70, Interleukin-12p70; IL-13, Interleukin-13; IL-16, Interleukin-16; IL-17, Interleukin-17; IL-18, Interleukin-18; IL-1b, Interleukin-1 beta; IL-1ra, Interleukin-1 receptor antagonist ; IL-2, Interleukin-2; IL-2ra, Interleukin-2 receptor antagonist; IL-4, Interleukin-4; IL-5, Interleukin-5; IL-6, Interleukin-6; IL-7, Interleukin-7; IL-8, Interleukin-8; IL-9, Interleukin-9; IP10, Interferon gamma-induced protein 10; MCP1, Monocyte chemotactic protein 1; MCP3, Monocyte chemotactic protein 3; MCSF, Macrophage colony stimulating factor; MIF, Macrophage migration inhibitory factor; MIG, Monokine induced by interferon (IFN)-gamma; MIP1a, Macrophage inflammatory protein 1-alpha; MIP1b, Macrophage inflammatory protein 1-beta; PDGFbb, Platelet derived growth factor bb; RANTES, Regulated upon activation normal T cell expressed and presumably secreted; SCF, Stem cell factor; SCGFb, Stem cell growth factor-beta; SDF1a, Stromal cell-derived factor-1alpha; TNFa, Tumor necrosis factor-alpha; TNFb, Tumor necrosis factor-beta; TRAIL, Tumor necrosis factor-related apoptosis-inducing ligand; VEGF, Vascular endothelial growth factor.

**Figure 4:**
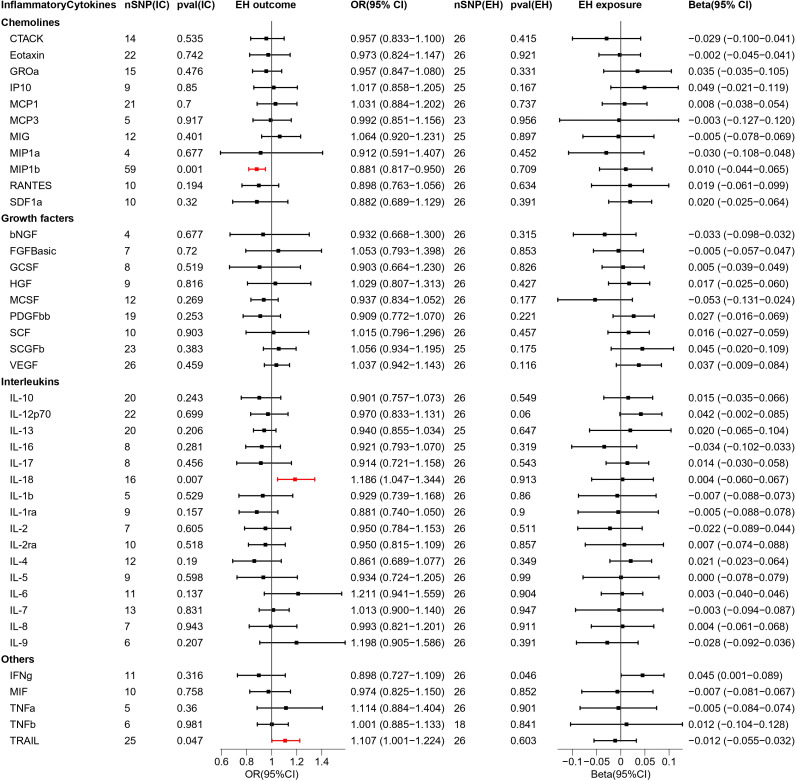
Causal estimations between 41 inflammatory cytokines and EH. The odds ratio (OR) was estimated using the random effect IVW method. The horizontal bars represent 95% confidence intervals (CI). SNP, Single nucleotide polymorphism; IC, Inflammatory Cytokine; pval, P-value; EH, Pre-existing hypertension complicating pregnancy, childbirth and the puerperium; OR, Odds ratio; CI, Confidence interval; CTACK, Cutaneous T cell-attracting chemokine; bNGF, Basic-nerve growth factor; FGFBasic, Basic fibroblast growth factor; GCSF, Granulocyte colony-stimulating factor; GROa, Gastroesophageal resuscitative occlusion of the aorta; HGF, Hepatocyte growth factor; IFNg, Interferon-gamma; IL-10, Interleukin-10; IL-12p70, Interleukin-12p70; IL-13, Interleukin-13; IL-16, Interleukin-16; IL-17, Interleukin-17; IL-18, Interleukin-18; IL-1b, Interleukin-1 beta; IL-1ra, Interleukin-1 receptor antagonist ; IL-2, Interleukin-2; IL-2ra, Interleukin-2 receptor antagonist; IL-4, Interleukin-4; IL-5, Interleukin-5; IL-6, Interleukin-6; IL-7, Interleukin-7; IL-8, Interleukin-8; IL-9, Interleukin-9; IP10, Interferon gamma-induced protein 10; MCP1, Monocyte chemotactic protein 1; MCP3, Monocyte chemotactic protein 3; MCSF, Macrophage colony stimulating factor; MIF, Macrophage migration inhibitory factor; MIG, Monokine induced by interferon (IFN)-gamma; MIP1a, Macrophage inflammatory protein 1-alpha; MIP1b, Macrophage inflammatory protein 1-beta; PDGFbb, Platelet derived growth factor bb; RANTES, Regulated upon activation normal T cell expressed and presumably secreted; SCF, Stem cell factor; SCGFb, Stem cell growth factor-beta; SDF1a, Stromal cell-derived factor-1alpha; TNFa, Tumor necrosis factor-alpha; TNFb, Tumor necrosis factor-beta; TRAIL, Tumor necrosis factor-related apoptosis-inducing ligand; VEGF, Vascular endothelial growth factor.

**Figure 5:**
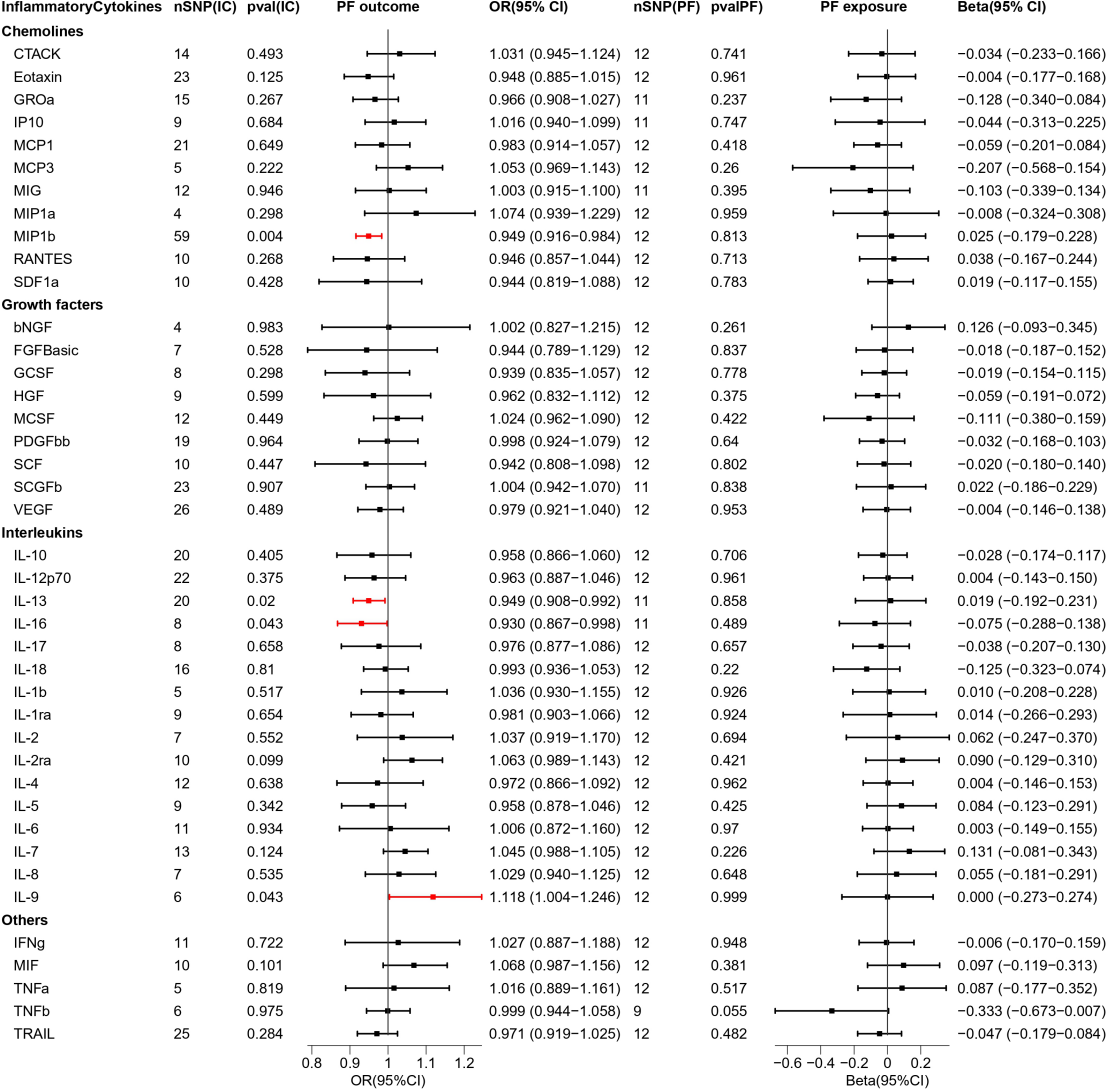
Causal estimations between 41 inflammatory cytokines and PF. The odds ratio (OR) was estimated using the random effect IVW method. The horizontal bars represent 95% confidence intervals (CI). SNP, Single nucleotide polymorphism; IC, Inflammatory Cytokine; pval, P-value; PF, Pre-eclampsia or poor fetal growth; OR, Odds ratio; CI, Confidence interval; CTACK, Cutaneous T cell-attracting chemokine; bNGF, Basic-nerve growth factor; FGFBasic, Basic fibroblast growth factor; GCSF, Granulocyte colony-stimulating factor; GROa, Gastroesophageal resuscitative occlusion of the aorta; HGF, Hepatocyte growth factor; IFNg, Interferon-gamma; IL-10, Interleukin-10; IL-12p70, Interleukin-12p70; IL-13, Interleukin-13; IL-16, Interleukin-16; IL-17, Interleukin-17; IL-18, Interleukin-18; IL-1b, Interleukin-1 beta; IL-1ra, Interleukin-1 receptor antagonist ; IL-2, Interleukin-2; IL-2ra, Interleukin-2 receptor antagonist; IL-4, Interleukin-4; IL-5, Interleukin-5; IL-6, Interleukin-6; IL-7, Interleukin-7; IL-8, Interleukin-8; IL-9, Interleukin-9; IP10, Interferon gamma-induced protein 10; MCP1, Monocyte chemotactic protein 1; MCP3, Monocyte chemotactic protein 3; MCSF, Macrophage colony stimulating factor; MIF, Macrophage migration inhibitory factor; MIG, Monokine induced by interferon (IFN)-gamma; MIP1a, Macrophage inflammatory protein 1-alpha; MIP1b, Macrophage inflammatory protein 1-beta; PDGFbb, Platelet derived growth factor bb; RANTES, Regulated upon activation normal T cell expressed and presumably secreted; SCF, Stem cell factor; SCGFb, Stem cell growth factor-beta; SDF1a, Stromal cell-derived factor-1alpha; TNFa, Tumor necrosis factor-alpha; TNFb, Tumor necrosis factor-beta; TRAIL, Tumor necrosis factor-related apoptosis-inducing ligand; VEGF, Vascular endothelial growth factor.

In this two-sample MR study, we investigated the correlation of each of the 4 types of HDP with 41 biomarkers—including interleukins, growth factors, and chemokines— thereby demonstrating that the inflammatory cytokines variably impact each disease type. Interleukin-9 (IL-9), macrophage migration inhibitory factor (MIF), MIP1b, interleukin-13 (IL-13), interleukin-16 (IL-16), and tumor necrosis factor-related apoptosis-inducing ligand (TRAIL) in PE; MIF and vascular endothelial growth factor (VEGF) in GH; interleukin-18 (IL-18), TRAIL, and MIP1b in EH; and IL-9, MIP1b, IL-13 and IL-16 in PF may serve as potential upstream factors for disease when inflammatory cytokines are used as exposures and disease is used as an outcome. In addition, VEGF, interleukin-12p70 (IL-12p70), interleukin-1 beta (IL-1b), interleukin-1 receptor antagonist (IL-1ra), interleukin-4 (IL4), interleukin-7 (IL-7), interleukin-8 (IL-8), macrophage colony stimulating factor (MCSF) in PE and macrophage inflammatory protein 1-alpha (MIP1a), hepatocyte growth factor (HGF), IL-18, IL-8 in GH might cause alterations in the expression levels of these cytokines through the pathogenic pathway if the disease type is used as exposure. Further, there is no reciprocal causal association between individual biomarkers and diseases under each disease type. The results of bidirectional MR analysis among the four types of HDP and inflammatory cytokines are presented in **Figures 2**–**5**.

### 3.1 Bidirectional interactions between 41 inflammatory cytokines and PE

The results of bidirectional MR analysis of inflammatory cytokines correlated with PE are depicted in **Figure 2**. When 41 inflammatory cytokines were used as exposures, 6 inflammatory factors were causally associated with the pathogenesis of PE disease, including IL9 (odds ratio (OR) = 1.168, 95%CI = 1.027–1.327, p = 0.018) and MIF (OR = 1.120, 95% CI = 1.021–1.228, p = 0.016) as facilitators, MIP1b (OR = 0.957, 95% CI = 0.917–0.998, p = 0.042), IL13 (OR = 0.947, 95% CI = 0.898–0.997, p = 0.039), IL16 (OR = 0.916, 95% CI = 0.843–0.996, p = 0.039), and TRAIL (OR = 0.913, 95% CI = 0.850–0.981, p = 0.013) playing inhibitory roles. Scatter plots were used to demonstrate the trends of inflammatory cytokines under the five MR methods (**Figure S1**).

When PE was used as exposure, the remaining 33 SNPs were involved in the MR analysis. The results are depicted in **Figure 2**. Eight inflammatory cytokines were affected by PE, among which the growth factor MCSF (Beta = -0.159, 95% CI= -0.280–0.037, p = 0.01) was negatively regulated, while the others were positively regulated. The remaining seven inflammatory factors were VEGF (Beta = 0.098, 95% CI = 0.026–0.170, p = 0.008), IL12p70 (Beta = 0.076, 95% CI = 0.009–0.143, p = 0.027), IL1b (Beta = 0.137, 95% CI = 0.032–0.243, p = 0.011), IL1ra (Beta = 0.131, 95% CI = 0.018–0.245, p = 0.023), IL4 (Beta = 0.086, 95% CI = 0.015–0.156, p = 0.017), IL7 (Beta = 0.109, 95% CI = 0.006–0.213, p = 0.039), IL8 (Beta = 0.125, 95% CI = 0.017–0.232, p = 0.023). The scatter plot of the above inflammatory cytokines is presented in **Figure S2**.

### 3.2 Bidirectional interactions between 41 inflammatory cytokines and GH

As 41 inflammatory cytokines were used for MR analysis as exposure, MIF (OR = 1.142, 95% CI = 1.029–1.267, p = 0.012), which acted as a promoter, and VEGF (OR = 0.950, 95% CI = 0.904–0.9995, p = 0.048), which acted as an inhibitor, were identified. The scatter plot is depicted in **Figure S3**.

Next, we reversed the exposure and outcome. MR analysis revealed that four inflammatory cytokines were screened out and all of them had increased expression under the influence of GH. The four cytokines are MIP1a (Beta = 0.122,95% CI = 0.024–0.219, p = 0.015) HGF (Beta = 0.070, 95% CI = 0.006–0.134, p = 0.032), IL-18 (Beta = 0.136, 95% CI = 0.030–0.241, p = 0.012), and IL-8 (Beta = 0.116, 95% CI = 0.018–0.213, p = 0.02). The scatter plot is depicted in **Figure S4**. In addition, the 95% CIs for GROa and SCGFb did not pass through 0 (**Figure 3**). The beta values for MR Eggar were in the opposite direction as that of IVW; thus, GROa (IVW beta = 0.106, MR Eggar beta = -0.143) and SCGFb (IVW beta = 0.129, MR Eggar Beta = -0.263) were not considered as statistically significant cytokines.

### 3.3 Bidirectional interactions between 41 inflammatory cytokines and EH

We explored the causal association of 41 inflammatory cytokines with EH as outcome and obtained three inflammatory cytokines with causal potential—IL-18 (OR = 1.186, 95% CI = 1.047–1.344, p = 0.007) and TRAIL (OR = 1.107, 95% CI = 1.001–1.224, p = 0.047) factors produced positive regulatory effects, while MIP1b (OR = 0.881, 95% CI = 0.817–0.950, p = 0.001) factor exerted negative effects. The scatter plot is depicted in **Figure S5**.

When exploring the MR analysis of the role of EH as exposure, only the IFNg was revealed as a statistically significant responder by the IVW method. However, the MR Eggar method obtained results with the opposite trend to that of the former; thus, the IFNg (IVW beta = 0.045, MR Eggar beta = -0.004) was eliminated.

### 3.4 Bidirectional interactions between 41 inflammatory cytokines and PF

When MR analysis was performed with 41 inflammatory cytokines as exposure and PF as outcome, one facilitator, IL-9 (OR = 1.118, 95% CI = 1.004–1.246, p = 0.043), and three inhibitory factors, MIP1b (OR = 0.949, 95% CI = 0.916–0.984, p = 0.004), IL-13 (OR = 0.949, 95% CI = 0.908–0.992, p = 0.02), and IL-16 (OR = 0.930, 95% CI = 0.867–0.998, p = 0.043) were found. The scatterplot is presented in **Figure S6**.

Reversing exposure and outcome, no significant inflammatory cytokines were found.

### 3.5 Sensitivity analysis

The results of the tests for heterogeneity and pleiotropy when inflammatory cytokines were used as exposures are presented in **Table S2**. The results of the tests for heterogeneity and pleiotropy when inflammatory cytokines were used as outcomes are presented in **Table S4**. In addition, the leave-one-out analysis results are presented in **Figure S7-12**. Details of SNPs when inflammatory cytokines were used as exposures are presented in **Table S3**. Details of SNPs when inflammatory cytokines were used as outcomes are presented in **Table S5**.

## 4 Discussion

Recently, numerous studies that explore the association of HDP with the immune system and the inflammatory cytokines produced by immune cells have been published. For example, TH17 cells and their secreted interleukin-17 (IL-17) levels are found to be increased in PE patients; TH17 cells induce pre-eclampsia by participating in placental ischemia, oxidative stress, and other pathways (23). It has also been suggested that VEGF may be an important biomarker for pre-eclampsia and has significant value in predicting its severity (24, 25). Despite the rich results of previous observational studies, the causal association among numerous important inflammatory cytokines with HDP has not been established due to the limitations of traditional epidemiology (26). Observational studies often provide biased associations due to the presence of confounding factors or reverse causality, thereby making it difficult to obtain credible conclusions using this method (27). In terms of pregnancy disorders, it is difficult to identify the causes of changes in inflammatory cytokine levels in patients with HDP through general observational studies. The presence of the disease itself, side effects caused by therapeutic drugs, underlying pathological immune response, the state of hidden infection, and harmful lifestyle habits (such as alcohol abuse and smoking) can cause changes in the levels of inflammatory cytokines (28–30). Recently, it has been reported that cytokine storms are highlighted as a common feature in pre-eclampsia and severe forms of coronavirus disease 2019 disease (31).

Our study found that more inflammatory cytokines exhibited significant changes in the initiation and progression of PE compared to GH. Among the four types of HDP, the inflammatory cytokines that recurrently appear upstream of the disease are IL-9, MIF, MIP1b, IL-13, IL-16, and TRAIL. The IL-9/IL-9R pathway is involved in regulating trophectoderm function, and increased IL-9 levels were found to result in increased tissue proliferation and invasiveness (32). IL-9 and IL-9R increase fibroblast proliferation as well as induce pro-inflammatory cytokines and metalloproteinases, which are key pathological processes that promote the formation of vascular opacities (33). Prior studies have suggested that Th9 cells may be involved in immune-mediated diseases such as allergy and autoimmune inflammatory diseases (34). Th1 or Th17 cells can induce CD4+IL-9+ T cell differentiation by promoting factors such as IL-1b and IL-12 (35). Under condition of TGF-β presence, Th-17 cells can produce IL-9 to participate in pro-inflammatory response (36). At present, research on Th9 cells in human pregnancy is limited, and the possible role of Th9 cells in preeclampsia needs to be further investigated. In addition, MIF is involved in multiple biophysiological processes that contribute to cell proliferation and differentiation, innate immune responses, and angiogenic biological activities (37). Previous studies have confirmed that large amounts of pro-inflammatory mediators are produced by MIF stimulation, including cytokines tumor necrosis factor-alpha (TNFa), IL-1b, interleukin-6 (IL-6), interferon-gamma (IFNg), matrix metalloproteinases, and nitric oxide (38, 39). Circulating concentrations of MIF are elevated in infected patients, inflammatory conditions, and autoimmune diseases (40). Therefore, MIF is recognized as a biomarker or pharmacological target for different diseases (41, 42), and MIF inhibitors might have considerable therapeutic benefit for numerous inflammatory and autoimmune diseases (43–46). Similar conditions have been found in mothers with pathological pregnancies (preterm birth (47) and pre-eclampsia (48). MIP1b was revealed to have the potential to recruit macrophages and dendritic cells (49, 50), which have the ability to secrete a range of cytokines/chemokines and enzymes implicated in angiogenesis and tissue remodeling (51). Furthermore, it has been demonstrated that macrophages promote trophoblastic invasion in pregnant mice (52). IL-13 exerts its anti-inflammatory effects by binding to IL-4 receptor alpha (IL-4ra) (53). In addition, IL-13 can downregulate the expression of IL-1b, IL-8, TNFa, and MIP1a (54–56). The anti-inflammatory potential of IL-13 has been therapeutically investigated in a variety of pathologies, including psoriasis, arthritis, and Alzheimer’s disease (57–59). Therefore, IL13 may be a key therapeutic target in pre-eclampsia. Currently, there are very few studies that investigate the correlation between IL-16 and HDP. It has been shown that IL-16 can initiate and amplify inflammatory responses through the release of cell signaling molecules that bind to CD4+ T cells (60). It is worth noting that Andrea L et al. found a noticeable decrease of IL-16 expression in hemolysis, elevated liver enzymes, and low platelet count (HELLP) syndrome (61). Due to the lack of clarity regarding how IL-16 is implicated in the etiopathogenesis of pre-eclampsia suggests, it is noteworthy that future research on IL-16 in such disorders is necessary. Tumor necrosis factor-related apoptosis-inducing ligand (TRAIL) is part of the TNF ligand family, and TRAIL and its receptor play essential roles in trophoblastic immunity and invasion during early pregnancy (62, 63). TRAIL induces apoptosis in vascular endothelial cells and vascular smooth muscle cells during the process of remodeling the uterine spiral arteries (64). It has recently been demonstrated that pregnant women who subsequently develop HDP have reduced plasma levels of TRAIL prior to 20 weeks gestation (65), thereby suggesting that TRAIL could serve as a new predictive, noninvasive biomarker for pregnant women with HDP.

Further, previous studies have revealed that IL-12p70, IL-1ra, IL-1b, IL-8, and IL-18 are considerably higher in PE group than in normal pregnant women (66, 67), which supports our findings. IL-1ra is elevated in PE, and elevated circulating IL-1ra reflect increased activity of the pro-inflammatory cytokines IL-1a and IL-1b, both of which are difficult to identify in serum levels because of their short circulating half-lives (68, 69). Moreover, it has been revealed that IL-1b and TNFa can significantly increase MCSF expression in early pregnancy decidual cells (70). IL-7 cytokine is necessary for the proliferation and survival of pathogenic Th17 cells, and has a promotional role in autoimmune diseases such as experimental autoimmune encephalomyelitis (71, 72).. In addition, previous evidence suggests that Th17 cells are observed to be increased in the decidual cells of patients with pre-eclampsia (73). IL-17 is a major effector molecule of Th17 cells, which plays an important role in inflammatory response, and IL-17 also induces the production of factors such as IL-6 and IL-8. However, IL-17 was not a statistically significant factor in the results of this MR analysis. In a meta-analysis published in 2020, the authors found no evidence that circulating IL-17 levels differed between pre-eclampsia and controls (74). Furthermore, Brewster et al. found that IL-17 levels were lower in patients with early-onset pre-eclampsia and higher in patients with late-onset compared to controls (75). Consequently, we consider that the role of IL-17 in pre-eclampsia needs to be approached with caution and further studies are still needed. Karol Charkiewicz et al. demonstrated that HGF was expressed in the plasma of patients with PE at a higher level than that in healthy pregnant women (76).

Currently, the most effective way to deal with pre-eclampsia is the prompt delivery of the fetus and placenta (77). Symptoms of pre-eclampsia and eclampsia are usually greatly relieved when the fetus and placental tissue are delivered from the mother. In our study, circulating inflammatory cytokines were also no longer active in patients after delivery. Moreover, we found that IL-9, MIP1b, IL-13, and IL-16 might be associated with fetal dysplasia. Previous researches have suggested that IL-9 induces immune tolerance during pregnancy; if abnormalities occur, the fetus is no longer tolerated by the mother and has adverse outcomes for both the mother and fetus (78). MIP1b affects cytotoxicity and causes tissue damage in the developing fetal brain and is a potential mechanism for intrauterine fetal dysplasia (79). Fetal growth restriction (FGR) maternal IL-13 levels are lower than those in normal pregnancies (80). IL-13 is a Th2 cytokine with anti-inflammatory characteristics. IL-13 inhibits the production of IL-6, IL-8, TNFa, and IL-12 as well as suppresses cytotoxicity and blocks pathologic inflammation (81). Lower levels of IL13 in FGR may indicate a more pronounced Th1 bias or pro-inflammatory cytokine bias. Denihan et al. investigated that IL-16 participated in the process of fetal brain injury and that its level associated with the long-term prognosis of neonatal hypoxic-ischemic encephalopathy (82).

For the first time, we adopted bidirectional two-sample MR analysis to explore the causal association between circulating inflammatory cytokines and the four types of HDP. Additionally, attention has been focused on inflammatory cytokines in pre-eclampsia that may contribute to poor fetal growth and development. Prior literature reviewed upstream and downstream inflammatory cytokines in HDP, thereby providing strong evidence for our results. Our study obtained different inflammatory cytokine profiles for both the incidence and progression of the disease, which offers new directions in the prevention, surveillance, and treatment of HDP. The pathogenesis and pathophysiology of HDP, represented by pre-eclampsia, are very complex and multifactorial. This includes endothelial dysfunction, oxidative stress, angiogenesis, neutrophil activation, anti-angiogenic factors, and syncytiotrophoblast microparticles, which are all involved in the pathophysiologic process of pre-eclampsia. Inflammatory cytokines play an important role in disease progression, but not all. This study only included 41 inflammatory cytokines, which is obviously a limitation. With in-depth study of HDP, more cytokines will be explored by researchers in the future and bring new perspectives and insights for early detection and therapeutic strategies of HDP.

There are several strengths of this study. First, genetic variation is randomly distributed during gamete formation and conception; thus, MR analysis avoids the limitations of observational studies (confounding, regression dilution, and reverse causation bias) (11). Second, we used separate samples for the circulating inflammatory cytokines and HDP data, and two-sample data avoided bias from weak instrumental variables. Third, we included two large-scale cohorts were included for MR analysis, thereby applying a sufficiently large sample size to ensure generalizability of causality. Moreover, two sets of data were treated as exposure and outcome to perform bidirectional MR analysis, respectively. Finally, so as to test the validity of the IV hypotheses, we performed sensitivity analyses, which included heterogeneity, pleiotropy, and leave-one-out analyses.

Nevertheless, there are also a few limitations of this study that must be discussed. First, the number of IVs as exposure varied for each inflammatory cytokine. During our analysis, a few IVs were discarded after screening; thus, the final MR analysis results could be influenced by the limited number of IVs. Despite this, we calculated statistical efficacy for each IV and excluded IVs with low efficacies in order to ensure that the results presented in this study remain reliable. Second, HDP occurs only in the female population, whereas circulating inflammatory cytokine GWASs are found in both male and female populations; thus, the presence of gender differences could potentially impact MR results. Third, this research data pertains to European populations; therefore, it needs to be treated with caution when generalizing to other populations. Finally, the immune system in pregnancy is complex and variable, with multiple changes in inflammatory cytokines influenced by a variety of factors, whereas it is difficult to dynamically monitor such a complex process in our study. Early-onset preeclampsia (onset < 34 weeks of gestation) and late-onset preeclampsia (onset ≥ 34 weeks of gestation), have different pathophysiological and immunopathological origins. However, limited by the availability of GWAS data, this study did not conduct subgroup analyses. With regard to the causal association of circulating inflammatory cytokines with HDP, deeper and more extensive studies are required in the future.

## Data availability statement

The datasets presented in this study can be found in online repositories. The names of the repository/repositories and accession number(s) can be found in the article/**Supplementary Material**.

## Author contributions

SG: Data curation, Formal Analysis, Investigation, Methodology, Project administration, Resources, Visualization, Writing – original draft, Writing – review & editing. XB: Conceptualization, Investigation, Methodology, Project administration, Resources, Supervision, Visualization, Writing – original draft, Writing – review & editing. JD: Formal Analysis, Investigation, Validation, Visualization, Writing – original draft, Writing – review & editing. RZ: Conceptualization, Investigation, Project administration, Resources, Supervision, Writing – original draft, Writing – review & editing.
