# Supplementary material for: Circulating inflammatory cytokines and hypertensive disorders of pregnancy: a two-sample Mendelian randomization study
Source: Front Immunol. 2023 Nov 16;14:1297929. doi: 10.3389/fimmu.2023.1297929 (PMC10687474; doi:10.3389/fimmu.2023.1297929)
Supplement: Supplementary file 1 [file DataSheet_1.docx]

Supplementary Material

Circulating inflammatory cytokines and hypertensive disorders of pregnancy: a two-sample Mendelian randomization study

Siqi Guan, Xiaoxu Bai, Jincheng Ding, Rujin Zhuang*,

*** Correspondence:** Rujin Zhuang: hmuzrj@163.com

# Supplementary Figures and Tables

## Supplementary Figures


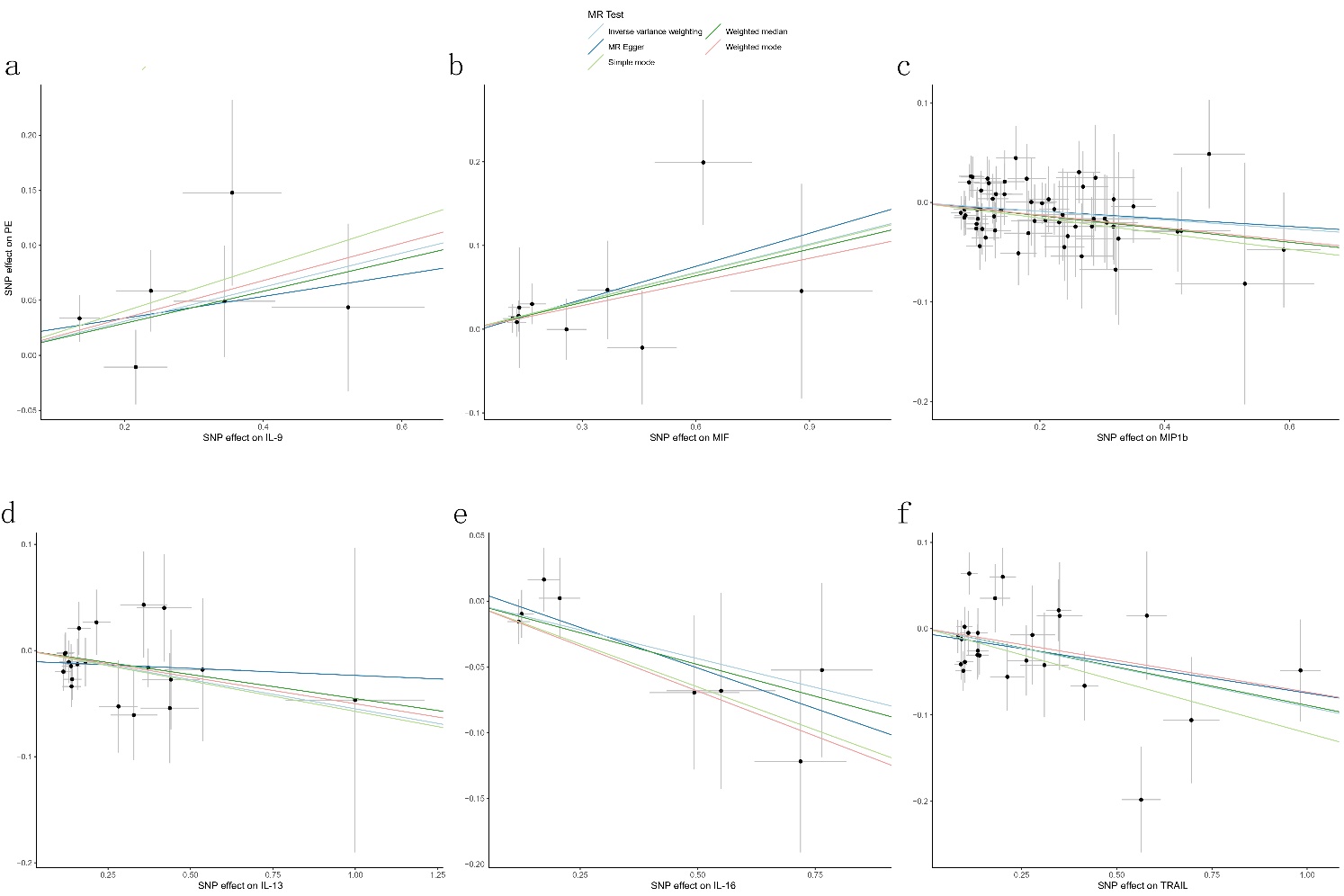


**Supplementary Figure 1.** Scatter plots of Mendelian randomization analyses for IL-9, MIF, MIP1b, IL-13, IL-16 and TRAIL (a-f) in PE. The slope of the lines represents the estimated causal effect of the MR methods.

Abbreviations: PE, Pre-eclampsia or eclampsia; SNP, Single nucleotide polymorphism; IL-9, Interleukin-9; MIF, Macrophage migration inhibitory factor; MIP1b, Macrophage inflammatory protein 1-beta; IL-13, Interleukin-13; IL-16, Interleukin-16; TRAIL, Tumor necrosis factor-related apoptosis-inducing ligand.


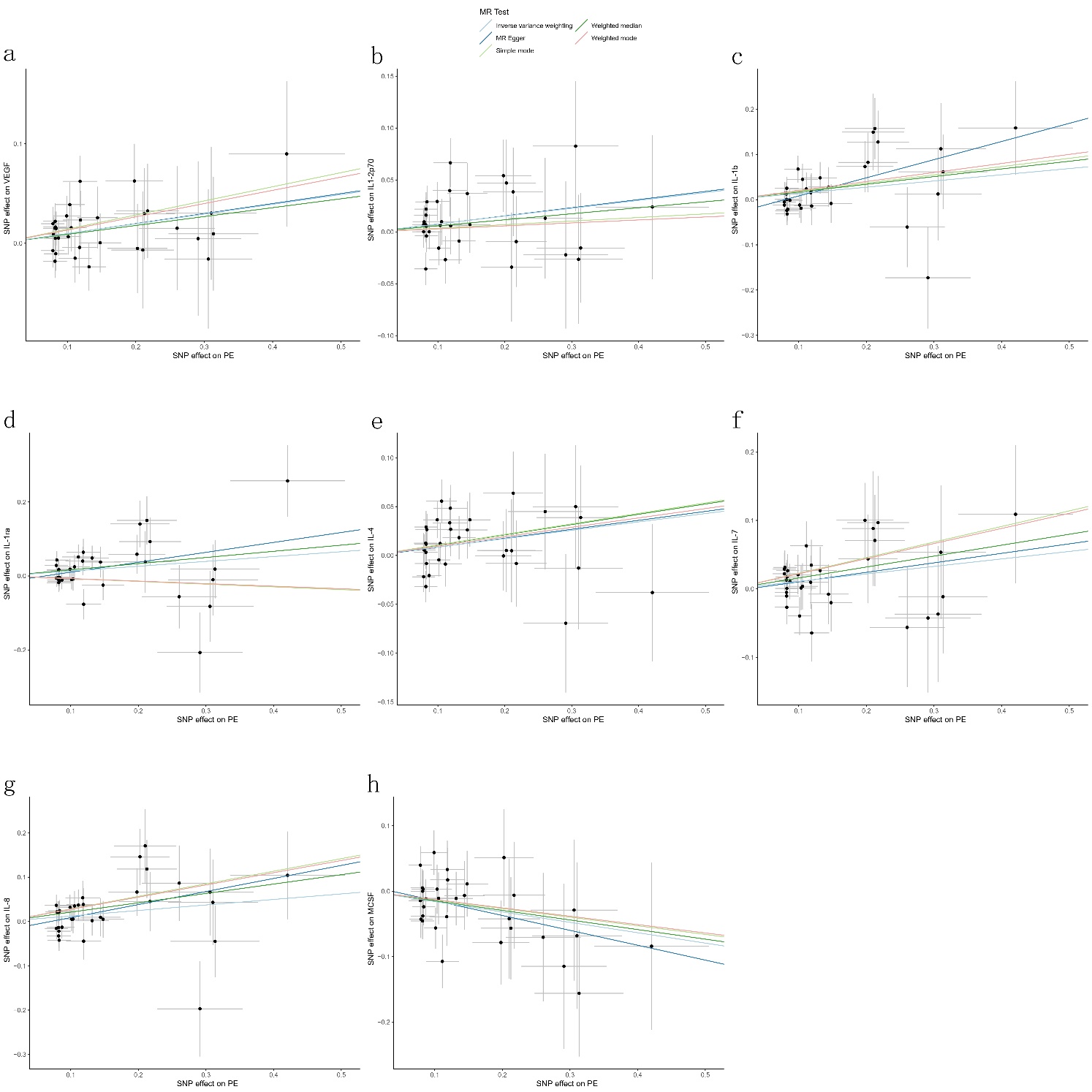


**Supplementary Figure 2.** Scatter plots of Mendelian randomization analyses between PE and inflammatory cytokines (a-h: VEGF, IL-12p70, IL-1b, IL-1ra, IL-4, IL-7, IL-8 and MCSF). The slope of the lines represents the estimated causal effect of the MR methods.

Abbreviations: PE, Pre-eclampsia or eclampsia; VEGF, Vascular endothelial growth factor; IL-12p70, Interleukin-12p70; IL-1b, Interleukin-1 beta; IL-1ra, Interleukin-1 receptor antagonist; IL-4, Interleukin-4; IL-7, Interleukin-7; IL-8, Interleukin-8; MCSF, Macrophage colony stimulating factor.


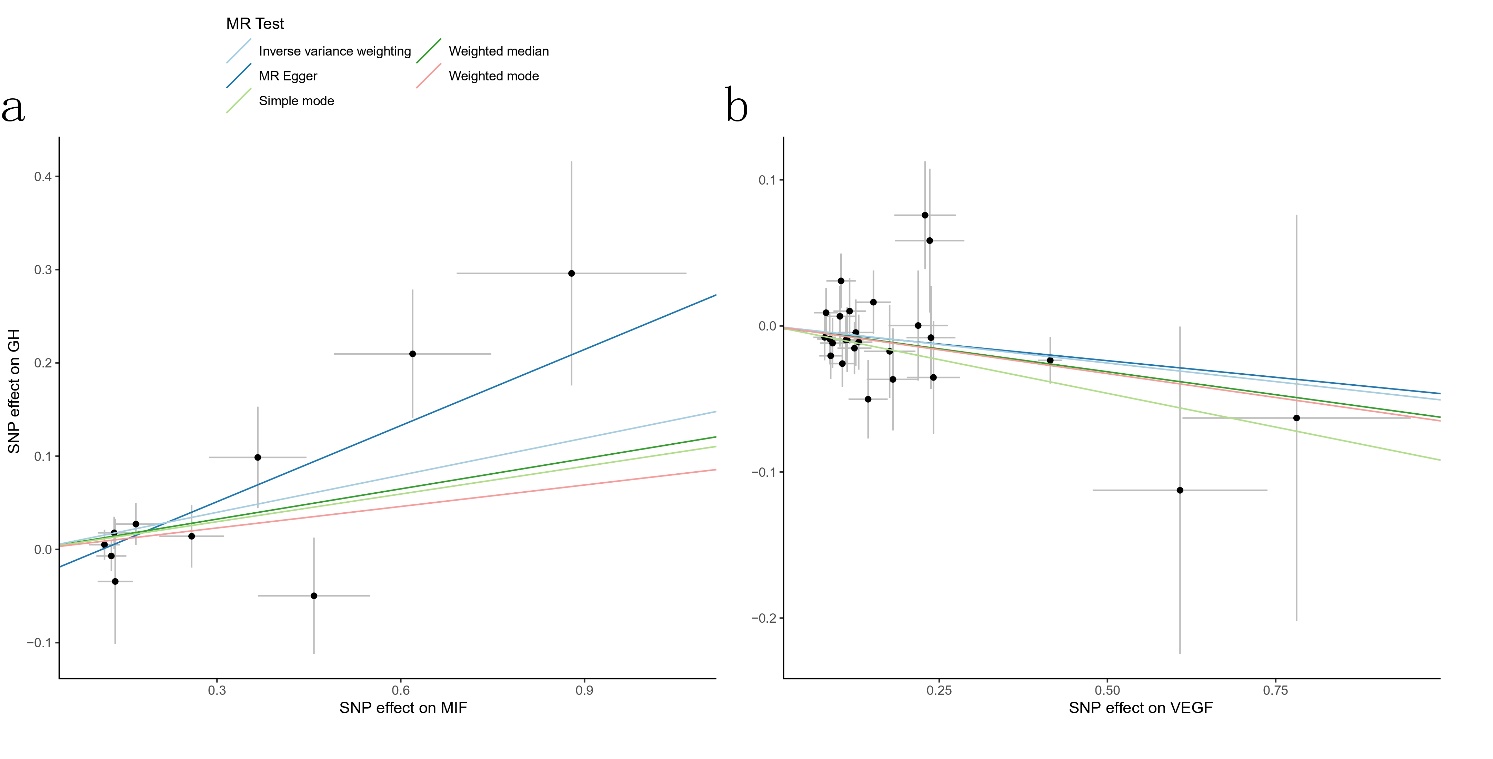


**Supplementary Figure 3.** Scatter plots of Mendelian randomization analyses for MIF and VEGF (a, b) in GH. The slope of the lines represents the estimated causal effect of the MR methods.

Abbreviations: GH, Gestational hypertension; MIF, Macrophage migration inhibitory factor; VEGF, Vascular endothelial growth factor.


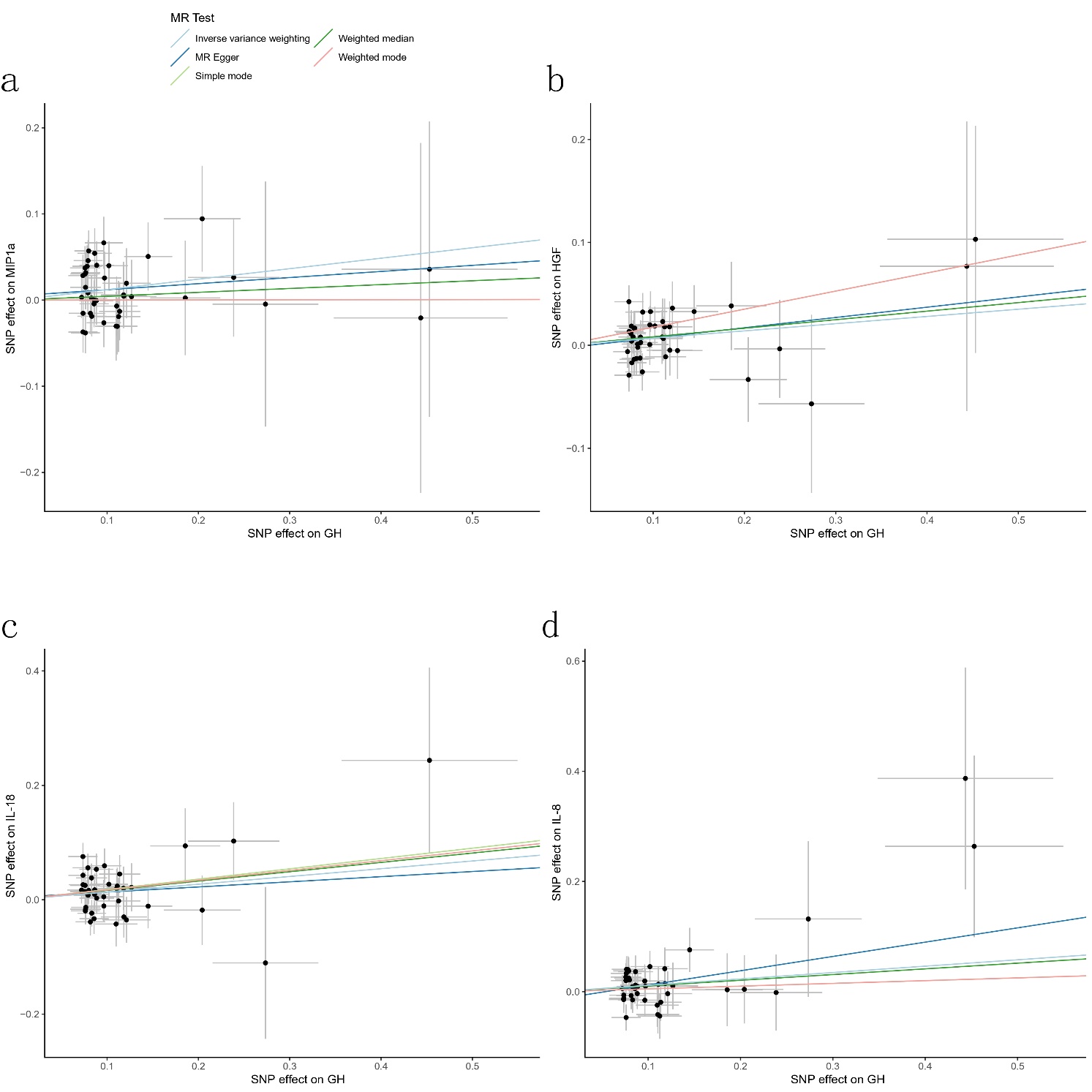


**Supplementary Figure 4.** Scatter plots of Mendelian randomization analyses between GH and inflammatory cytokines (a-d: MIP1a, HGF, IL-18 and IL-8). The slope of the lines represents the estimated causal effect of the MR methods.

Abbreviations: GH, Gestational hypertension; MIP1a, Macrophage inflammatory protein 1-alpha; HGF, Hepatocyte growth factor; IL-18, Interleukin-18; IL-8, Interleukin-8.


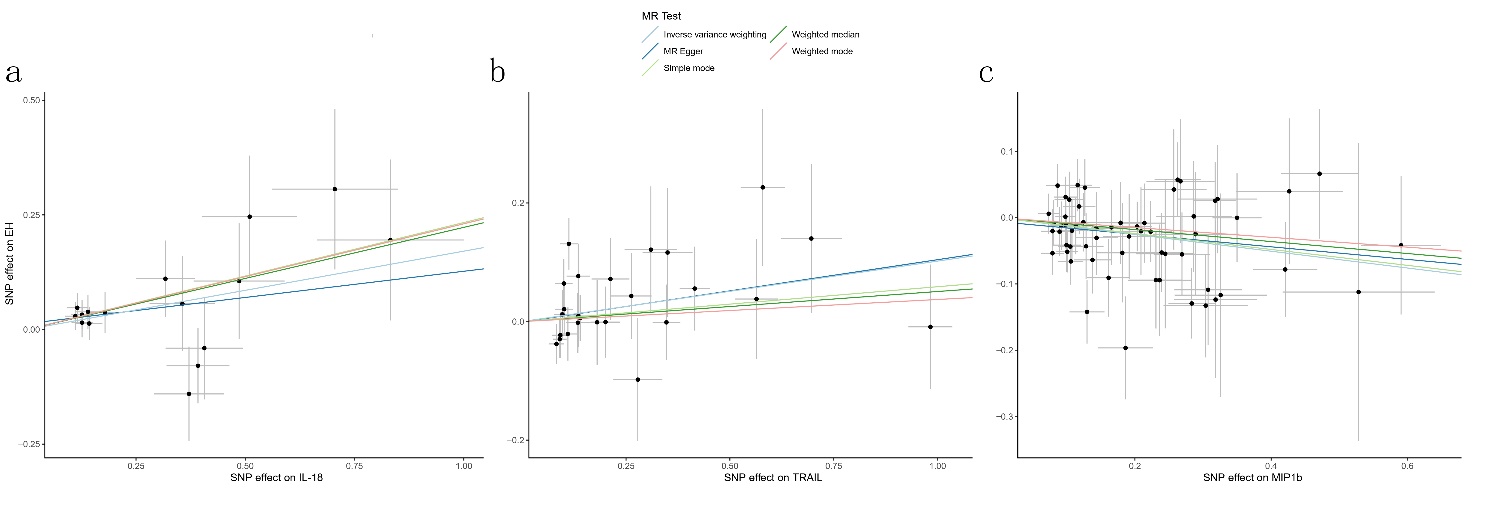


**Supplementary Figure 5.** Scatter plots of Mendelian randomization analyses for IL-18, TRAIL and MIP1b (a-c) in EH. The slope of the lines represents the estimated causal effect of the MR methods.

Abbreviations: EH, Pre-existing hypertension complicating pregnancy, childbirth and the puerperium; IL-18, Interleukin-18; TRAIL, Tumor necrosis factor-related apoptosis-inducing ligand; MIP1b, Macrophage inflammatory protein 1-beta.


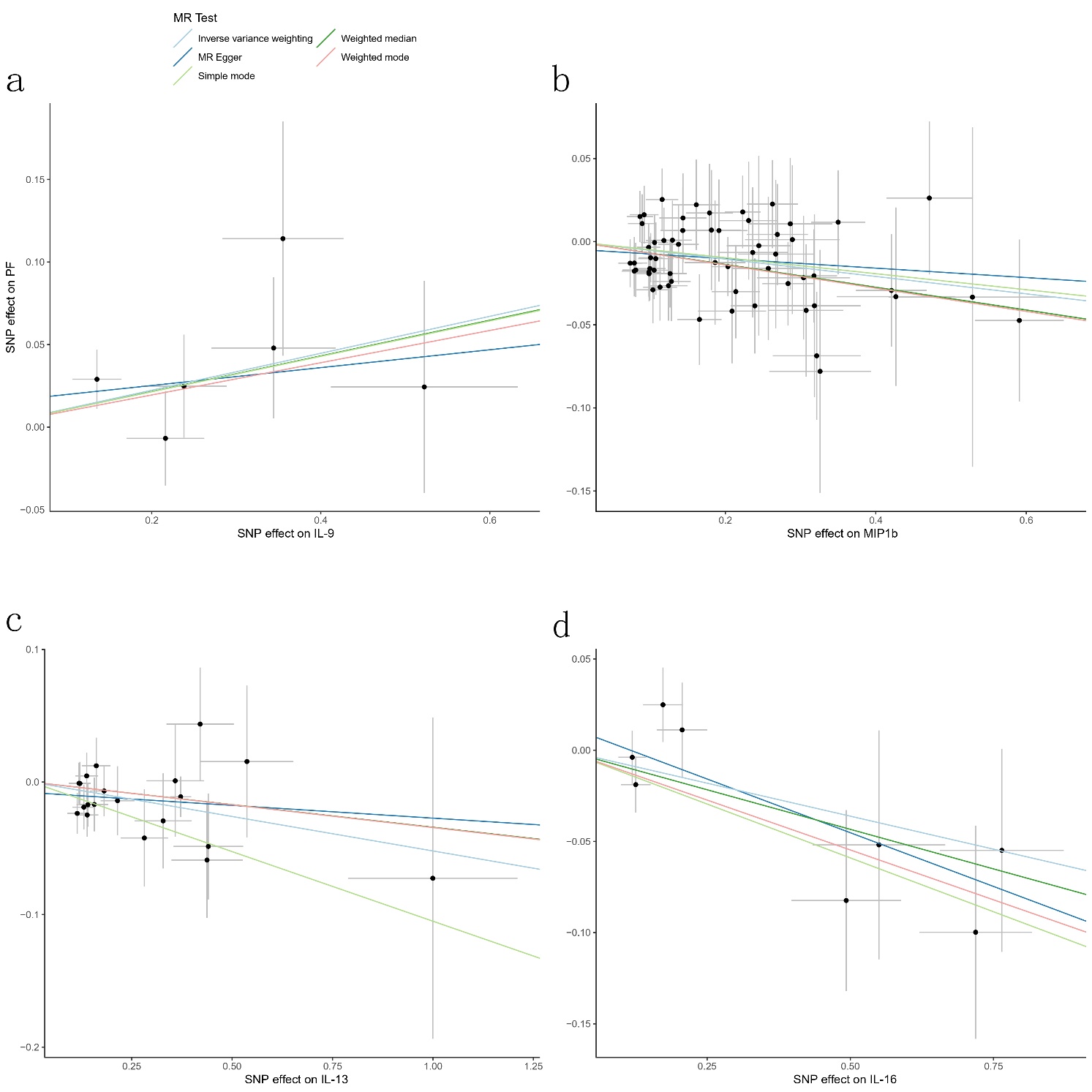


**Supplementary Figure 6.** Scatter plots of Mendelian randomization (MR) analyses for IL-9, MIP1b, IL-13 and IL-16 (a-d) in PF. The slope of the lines represents the estimated causal effect of the MR methods.

Abbreviations: PF, Pre-eclampsia or poor fetal growth; IL-9, Interleukin-9; MIP1b, Macrophage inflammatory protein 1-beta; IL-13, Interleukin-13; IL-16, Interleukin-16.


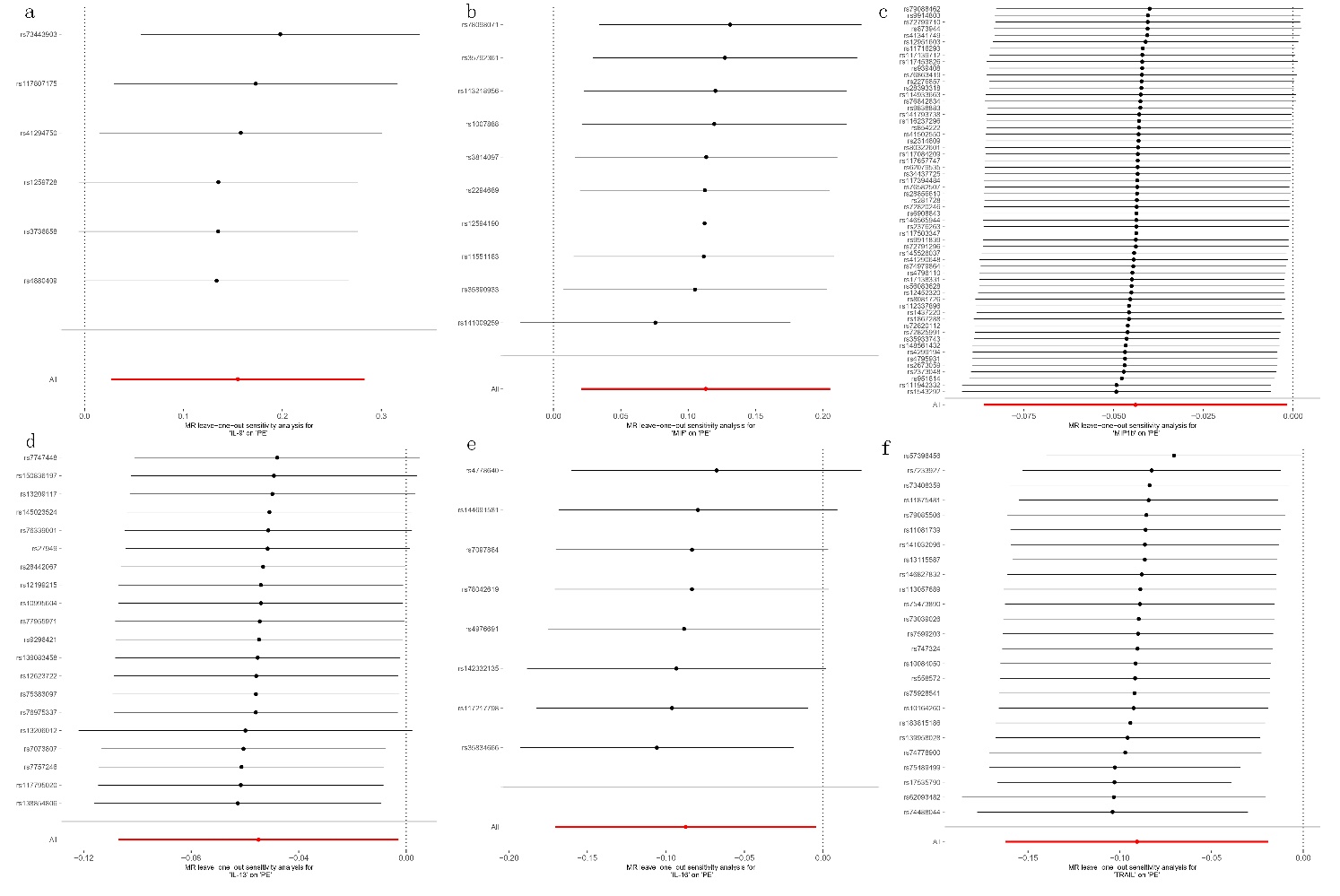


**Supplementary Figure 7.** Leave-one-out sensitivity analyses of Mendelian randomization analyses for IL-9, MIF, MIP1b, IL-13, IL-16 and TRAIL (a-f) in PE.

Abbreviations: PE, Pre-eclampsia or eclampsia; SNP, Single nucleotide polymorphism; IL-9, Interleukin-9; MIF, Macrophage migration inhibitory factor; MIP1b, Macrophage inflammatory protein 1-beta; IL-13, Interleukin-13; IL-16, Interleukin-16; TRAIL, Tumor necrosis factor-related apoptosis-inducing ligand.


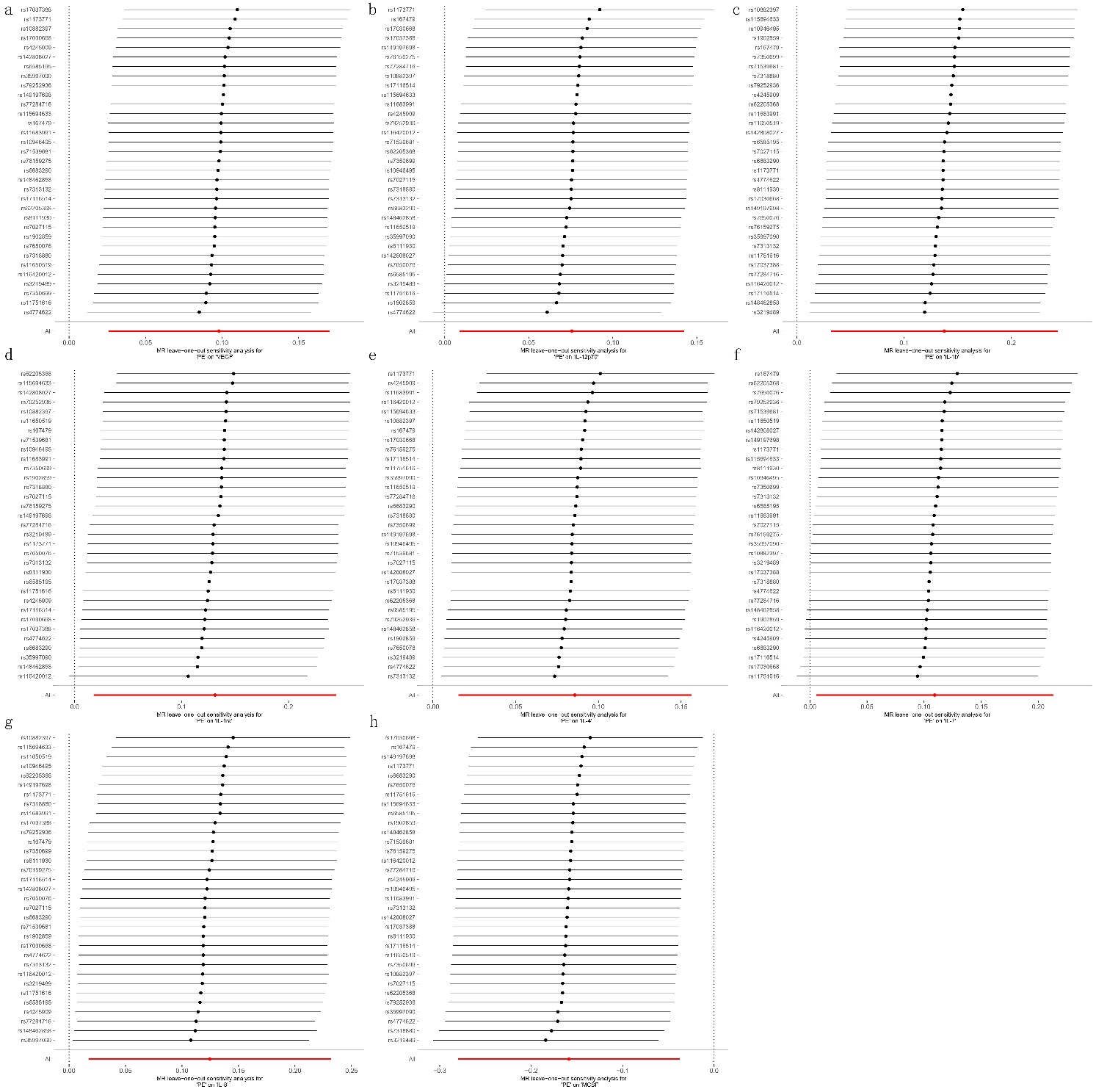


**Supplementary Figure 8.** Leave-one-out sensitivity analyses of Mendelian randomization analyses between PE and inflammatory cytokines (a-h: VEGF, IL-12p70, IL-1b, IL-1ra, IL-4, IL-7, IL-8 and MCSF).

Abbreviations: Abbreviations: PE, Pre-eclampsia or eclampsia; VEGF, Vascular endothelial growth factor; IL-12p70, Interleukin-12p70; IL-1b, Interleukin-1 beta; IL-1ra, Interleukin-1 receptor antagonist; IL-4, Interleukin-4; IL-7, Interleukin-7; IL-8, Interleukin-8; MCSF, Macrophage colony stimulating factor.


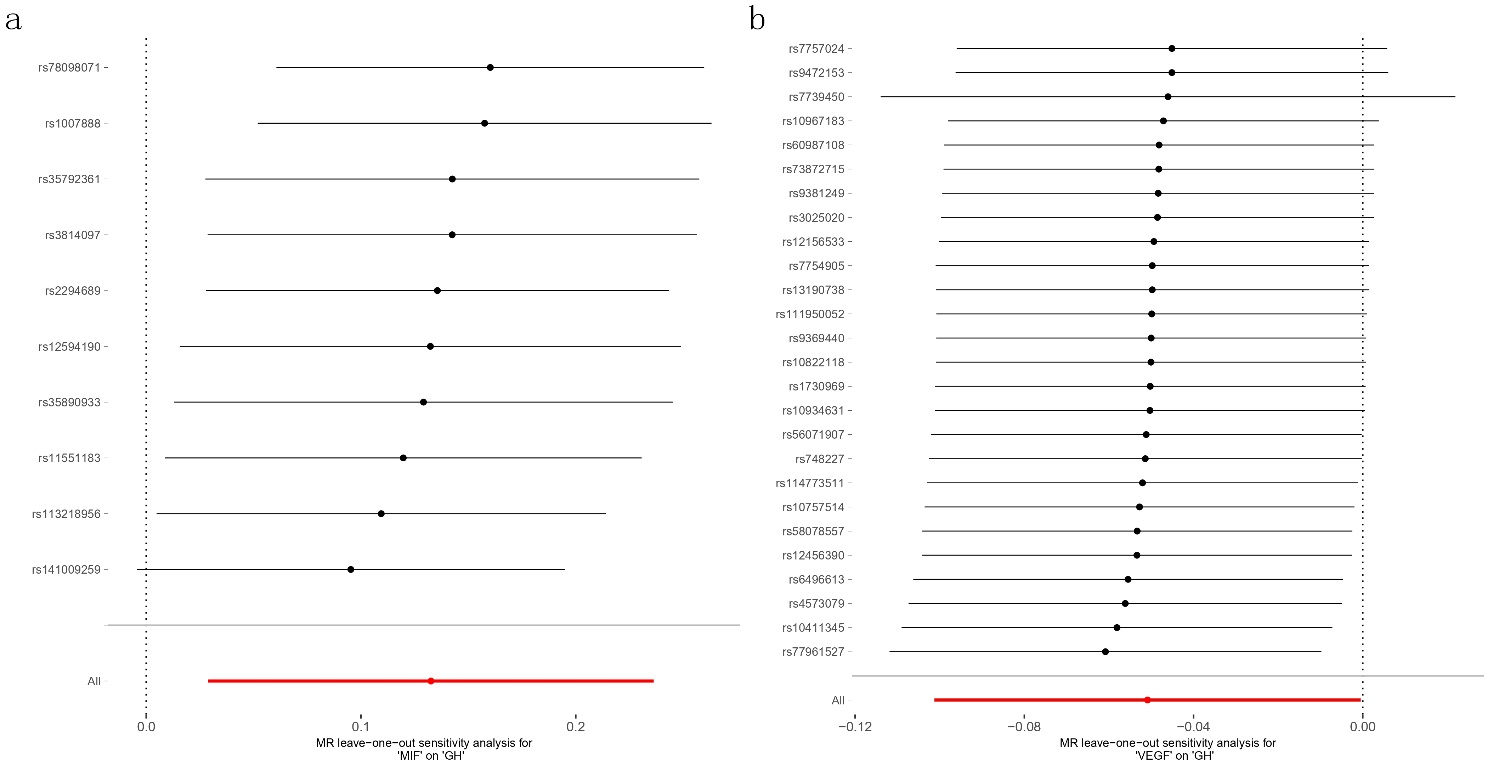


**Supplementary Figure 9.** Leave-one-out sensitivity analyses of Mendelian randomization analyses for MIF and VEGF (a, b) in GH.

Abbreviations: GH, Gestational hypertension; MIF, Macrophage migration inhibitory factor; VEGF, Vascular endothelial growth factor.


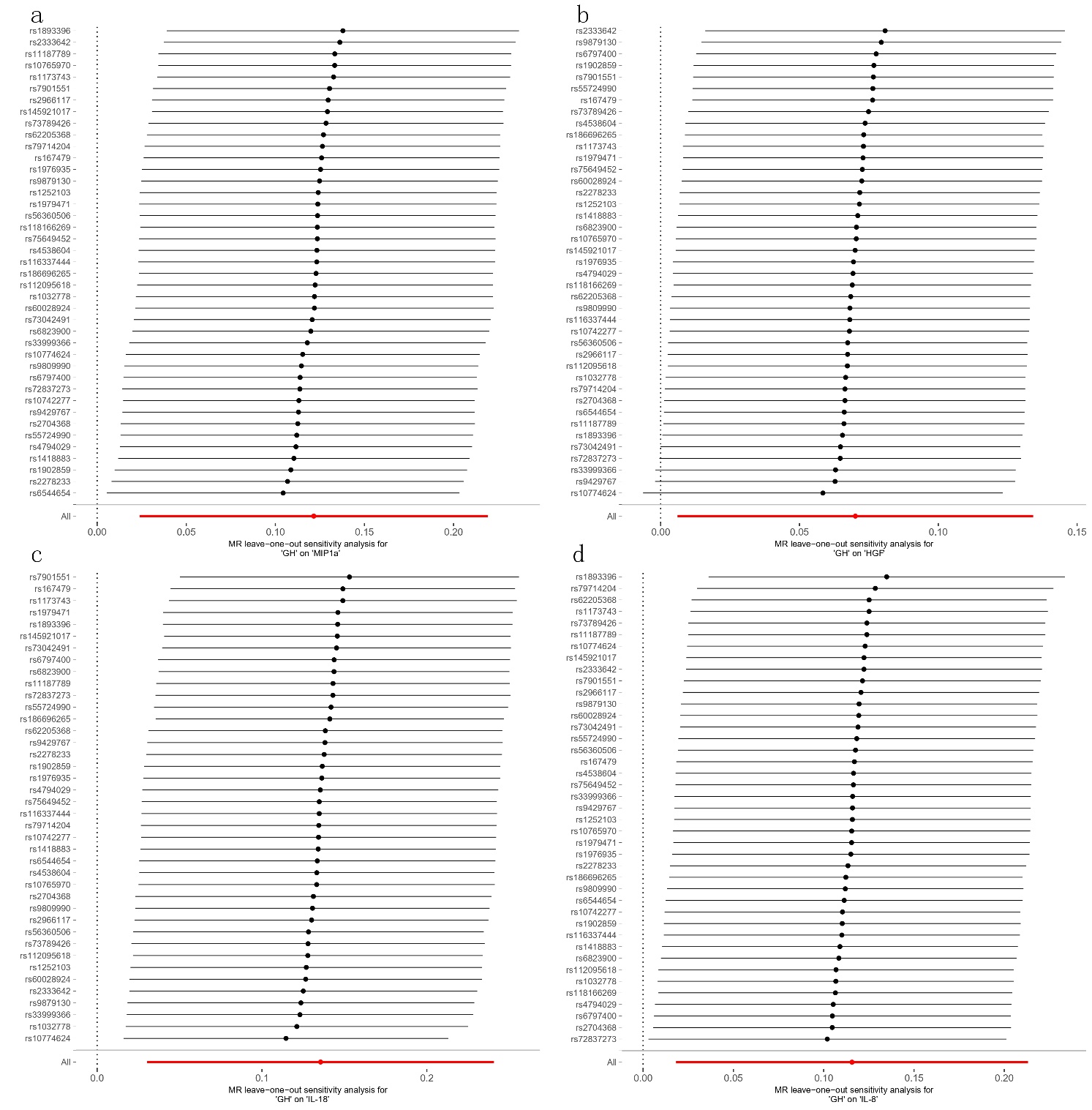


**Supplementary Figure 10.** Leave-one-out sensitivity analyses of Mendelian randomization analyses between GH and inflammatory cytokines (a-d: MIP1a, HGF, IL-18 and IL-8).

Abbreviations: GH, Gestational hypertension; MIP1a, Macrophage inflammatory protein 1-alpha; HGF, Hepatocyte growth factor; IL-18, Interleukin-18; IL-8, Interleukin-8.


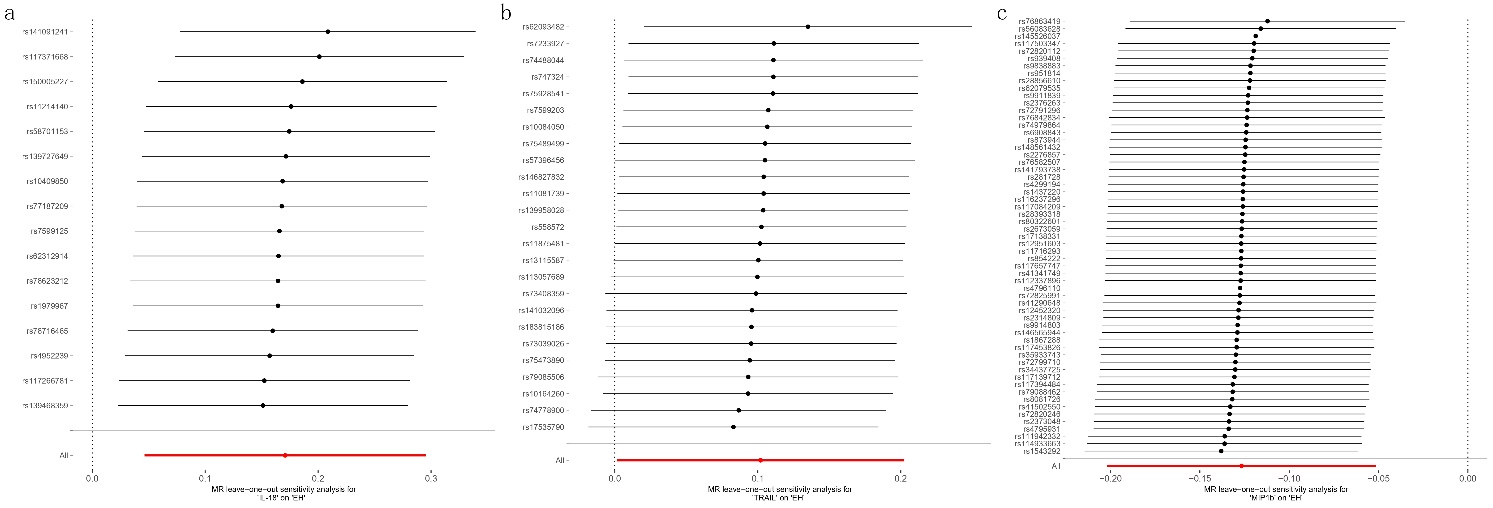


**Supplementary Figure 11.** Leave-one-out sensitivity analyses of Mendelian randomization analyses for IL-18, TRAIL and MIP1b (a-c) in EH.

Abbreviations: EH, Pre-existing hypertension complicating pregnancy, childbirth and the puerperium; IL-18, Interleukin-18; TRAIL, Tumor necrosis factor-related apoptosis-inducing ligand; MIP1b, Macrophage inflammatory protein 1-beta.


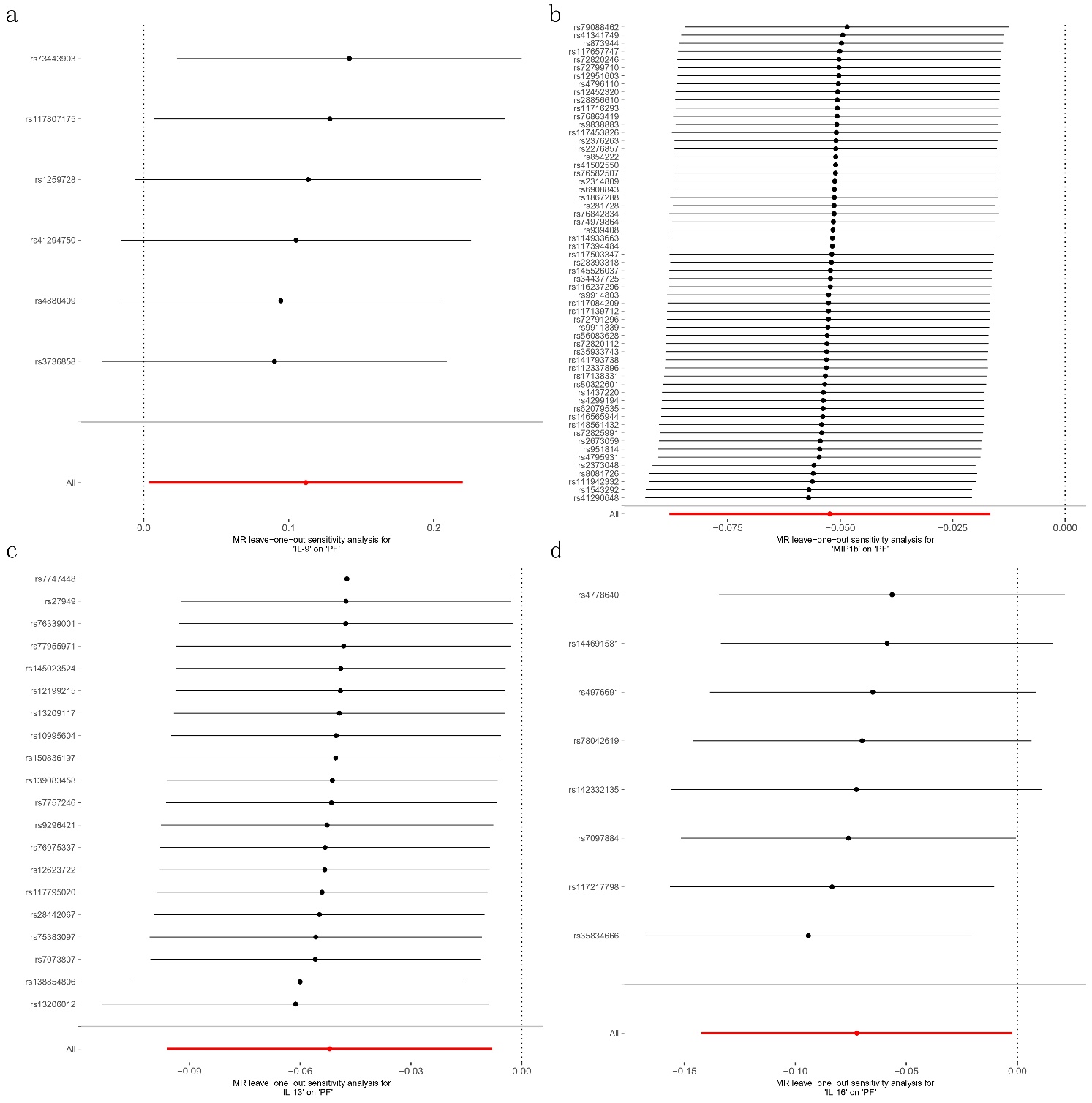


**Supplementary Figure 12.** Leave-one-out sensitivity analyses of Mendelian randomization analyses for IL-9, MIP1b, IL-13 and IL-16 (a-d) in PF.

Abbreviations: PF, Pre-eclampsia or poor fetal growth; IL-9, Interleukin-9; MIP1b, Macrophage inflammatory protein 1-beta; IL-13, Interleukin-13; IL-16, Interleukin-16.

## Supplementary Tables

**Supplementary Table 1.** Details of 4 FinnGen data for hypertensive disorders of pregnancy.

| **FinnGen** | **Type** | **FinnGen introduction** | **Median age**  **(years)** | **N-case** | **N-control** |
| --- | --- | --- | --- | --- | --- |
| PRE_OR_ECLAMPSIA | PE | Pre-eclampsia or eclampsia | 28.66 | 7212 | 194266 |
| GESTAT_HYPERT | GH | Gestational [pregnancy-induced] hypertension | 29.80 | 8502 | 194266 |
| EXIST_HYPERT_COMPLIC | EH | Pre-existing hypertension complicating pregnancy, childbirth and the puerperium | 32.96 | 2223 | 194266 |
| PREEC_OR_FETGRO | PF | Pre-eclampsia or poor fetal growth | 29.11 | 10297 | 200573 |

Abbreviations: N-case, number of case groups; N-control, number of control groups.

**Supplementary Table 2.** Heterogeneity and pleiotropy tests of the causal effects of inflammatory cytokines on HDP.

| **Exposure** | **Heterogeneity** | | **MR Eggar** | | **MR PRESSO** |
| --- | --- | --- | --- | --- | --- |
|  | **Cochran’s Q** | **Rucker’s Q** | **intercept** | **p-intercept** | **Global test p** |
| **PE** |  |  |  |  |  |
| MIP1b | 0.9925 | 0.9901 | -0.001 | 0.8844 | 0.989 |
| IL-13 | 0.8914 | 0.8898 | -0.0102 | 0.3963 | 0.904 |
| IL-16 | 0.8586 | 0.83 | 0.0107 | 0.5297 | 0.869 |
| IL-9 | 0.5701 | 0.4473 | 0.014 | 0.717 | 0.634 |
| MIF | 0.8026 | 0.7259 | -0.0041 | 0.814 | 0.778 |
| TRAIL | 0.0359 | 0.0291 | -0.0058 | 0.6239 | 0.748 |
| **GH** |  |  |  |  |  |
| VEGF | 0.659 | 0.6045 | -0.0011 | 0.8949 | 0.699 |
| MIF | 0.1528 | 0.3018 | -0.0305 | 0.1146 | 0.147 |
| **EH** |  |  |  |  |  |
| MIP1b | 0.7281 | 0.7033 | -0.006 | 0.6392 | 0.721 |
| IL-18 | 0.6424 | 0.5927 | 0.0127 | 0.5866 | 0.672 |
| TRAIL | 0.5618 | 0.5028 | -0.0009 | 0.957 | 0.577 |
| **PF** |  |  |  |  |  |
| MIP1b | 0.9937 | 0.9932 | -0.0046 | 0.4556 | 0.996 |
| IL-13 | 0.9424 | 0.9399 | -0.008 | 0.4291 | 0.945 |
| IL-16 | 0.4858 | 0.4856 | 0.0136 | 0.3545 | 0.528 |
| IL-9 | 0.6684 | 0.5614 | 0.0144 | 0.6594 | 0.714 |

Abbreviations: PE, Pre-eclampsia or eclampsia; MIP1b, Macrophage inflammatory protein 1-beta; IL-13, Interleukin-13; IL-16, Interleukin-16; IL-9, Interleukin-9; MIF, Macrophage migration inhibitory factor; TRAIL, Tumor necrosis factor-related apoptosis-inducing ligand; GH, Gestational hypertension; VEGF, Vascular endothelial growth factor; EH, Pre-existing hypertension complicating pregnancy, childbirth and the puerperium; IL-18, Interleukin-18;

**Supplementary Table 3.** Details of inflammatory cytokines predicting SNPs with hypertensive disease of pregnancy.

|  | **Exposure** | | | | | | **Outcome** | | |
| --- | --- | --- | --- | --- | --- | --- | --- | --- | --- |
| **SNP** | **Effect allele** | **Other allele** | **Beta** | **Se** | **Pval** | **F** | **Log(OR)** | **Se** | **Pval** |
| ***PE*** |  |  |  |  |  |  |  |  |  |
| **MIP1b** |  |  |  |  |  |  |  |  |  |
| rs111942332 | T | G | -0.471 | 0.057 | 1.52E-16 | 68.053 | -0.049 | 0.055 | 0.372 |
| rs112337896 | A | G | 0.289 | 0.063 | 4.4E-06 | 21.051 | 0.025 | 0.053 | 0.643 |
| rs114933663 | T | C | 0.318 | 0.031 | 3.71E-25 | 107.134 | -0.024 | 0.033 | 0.466 |
| rs116237296 | A | G | 0.528 | 0.112 | 2.15E-06 | 22.453 | -0.082 | 0.121 | 0.501 |
| rs117084209 | C | G | 0.245 | 0.043 | 1.14E-08 | 32.626 | -0.034 | 0.064 | 0.597 |
| rs117139712 | T | C | -0.267 | 0.050 | 9.38E-08 | 28.487 | 0.054 | 0.053 | 0.307 |
| rs11716293 | C | G | 0.099 | 0.019 | 1.69E-07 | 27.210 | -0.022 | 0.020 | 0.278 |
| rs117394484 | T | C | -0.427 | 0.079 | 5.73E-08 | 29.450 | 0.029 | 0.064 | 0.656 |
| rs117453826 | A | G | -0.591 | 0.059 | 1.53E-23 | 99.874 | 0.048 | 0.058 | 0.410 |
| rs117503347 | T | C | 0.304 | 0.062 | 9.60E-07 | 24.020 | -0.016 | 0.044 | 0.712 |
| rs117657747 | A | G | 0.209 | 0.045 | 4.01E-06 | 21.259 | -0.018 | 0.037 | 0.629 |
| rs12452320 | A | C | 0.214 | 0.032 | 2.09E-11 | 44.951 | 0.003 | 0.033 | 0.924 |
| rs12951603 | A | G | -0.113 | 0.023 | 4.67E-07 | 25.306 | 0.035 | 0.024 | 0.138 |
| rs141793738 | A | G | 0.182 | 0.039 | 3.15E-06 | 21.765 | -0.031 | 0.043 | 0.470 |
| rs1437220 | T | C | 0.144 | 0.032 | 4.92E-06 | 20.806 | 0.021 | 0.032 | 0.511 |
| rs145526037 | T | G | -0.186 | 0.041 | 4.47E-06 | 21.051 | 0.000 | 0.044 | 0.992 |
| rs146565944 | T | C | 0.286 | 0.056 | 2.90E-07 | 26.319 | -0.016 | 0.047 | 0.727 |
| rs148561432 | A | G | -0.269 | 0.041 | 3.75E-11 | 43.705 | -0.016 | 0.036 | 0.662 |
| rs1543292 | A | G | 0.263 | 0.034 | 7.34E-15 | 60.392 | 0.030 | 0.031 | 0.334 |
| rs17138331 | A | G | -0.143 | 0.030 | 1.13E-06 | 23.624 | -0.008 | 0.031 | 0.790 |
| rs1867288 | C | G | 0.203 | 0.022 | 2.67E-21 | 89.479 | -0.001 | 0.021 | 0.971 |
| rs2276857 | T | C | -0.128 | 0.026 | 5.7E-07 | 24.916 | 0.028 | 0.028 | 0.318 |
| rs2314809 | T | C | -0.074 | 0.016 | 2.9E-06 | 21.911 | 0.010 | 0.017 | 0.541 |
| rs2373048 | A | T | -0.116 | 0.022 | 6.93E-08 | 29.103 | -0.024 | 0.022 | 0.277 |
| rs2376263 | A | G | 0.105 | 0.019 | 1.79E-08 | 31.701 | -0.007 | 0.021 | 0.747 |
| rs2673059 | T | C | 0.092 | 0.019 | 1.68E-06 | 23.004 | 0.025 | 0.021 | 0.215 |
| rs281728 | A | C | -0.079 | 0.017 | 3.89E-06 | 21.338 | 0.007 | 0.019 | 0.704 |
| rs28393318 | A | G | -0.108 | 0.024 | 4.62E-06 | 20.960 | 0.027 | 0.026 | 0.302 |
| rs28856610 | T | C | -0.307 | 0.050 | 5.12E-10 | 38.556 | 0.020 | 0.047 | 0.669 |
| rs34437725 | T | C | -0.257 | 0.048 | 9.17E-08 | 28.563 | 0.024 | 0.051 | 0.635 |
| rs35933743 | T | G | -0.118 | 0.024 | 6.56E-07 | 24.701 | -0.019 | 0.024 | 0.410 |
| rs41290648 | A | G | 0.223 | 0.024 | 9.21E-21 | 87.116 | -0.007 | 0.026 | 0.793 |
| rs41341749 | A | G | -0.166 | 0.029 | 1.67E-08 | 31.719 | 0.051 | 0.032 | 0.114 |
| rs41502550 | T | C | 0.127 | 0.022 | 8.13E-09 | 33.107 | -0.014 | 0.024 | 0.560 |
| rs4299194 | T | C | 0.089 | 0.019 | 2.33E-06 | 22.369 | 0.026 | 0.021 | 0.209 |
| rs4795931 | A | G | -0.087 | 0.017 | 6.10E-07 | 24.994 | -0.020 | 0.018 | 0.269 |
| rs4796110 | A | G | 0.124 | 0.026 | 1.15E-06 | 23.608 | 0.004 | 0.025 | 0.882 |
| rs56083628 | T | C | -0.130 | 0.026 | 4.45E-07 | 25.463 | -0.008 | 0.028 | 0.767 |
| rs62079535 | A | G | 0.231 | 0.039 | 2.91E-09 | 35.224 | -0.020 | 0.042 | 0.635 |
| rs6908843 | A | G | 0.100 | 0.021 | 1.78E-06 | 22.751 | -0.007 | 0.023 | 0.750 |
| rs72791296 | T | C | 0.236 | 0.047 | 3.97E-07 | 25.729 | -0.012 | 0.046 | 0.789 |
| rs72799710 | T | C | -0.104 | 0.022 | 1.79E-06 | 22.831 | 0.044 | 0.024 | 0.065 |
| rs72820112 | T | C | 0.106 | 0.018 | 7.80E-09 | 33.480 | 0.012 | 0.020 | 0.542 |
| rs72820246 | T | G | -0.098 | 0.017 | 4.37E-09 | 34.639 | 0.007 | 0.017 | 0.704 |
| rs72825991 | A | G | -0.179 | 0.031 | 9.44E-09 | 32.907 | -0.024 | 0.035 | 0.496 |
| rs74979864 | A | T | -0.318 | 0.061 | 2.03E-07 | 26.972 | -0.003 | 0.065 | 0.961 |
| rs76582507 | A | G | 0.326 | 0.068 | 1.42E-06 | 23.236 | -0.036 | 0.087 | 0.675 |
| rs76842834 | T | C | -0.421 | 0.047 | 4.46E-19 | 79.762 | 0.029 | 0.040 | 0.467 |
| rs76863419 | T | G | -0.283 | 0.034 | 9.77E-17 | 69.005 | 0.024 | 0.030 | 0.419 |
| rs79088462 | T | C | -0.321 | 0.058 | 3.77E-08 | 30.280 | 0.068 | 0.046 | 0.138 |
| rs80322601 | T | C | -0.192 | 0.038 | 4.69E-07 | 25.390 | 0.019 | 0.036 | 0.609 |
| rs8081726 | T | C | -0.350 | 0.036 | 2.74E-22 | 94.445 | 0.004 | 0.037 | 0.916 |
| rs854222 | A | C | 0.081 | 0.018 | 4.95E-06 | 20.886 | -0.013 | 0.019 | 0.500 |
| rs873944 | T | C | 0.239 | 0.032 | 1.03E-13 | 55.170 | -0.045 | 0.034 | 0.188 |
| rs939408 | A | C | -0.101 | 0.016 | 3.01E-10 | 39.602 | 0.016 | 0.018 | 0.350 |
| rs951814 | A | G | 0.161 | 0.032 | 2.92E-07 | 26.247 | 0.045 | 0.032 | 0.166 |
| rs9838883 | T | C | 0.079 | 0.017 | 1.79E-06 | 22.643 | -0.015 | 0.018 | 0.397 |
| rs9911839 | T | G | -0.138 | 0.027 | 3.52E-07 | 26.004 | 0.007 | 0.028 | 0.794 |
| rs9914803 | T | C | 0.098 | 0.016 | 3.47E-10 | 39.455 | -0.026 | 0.017 | 0.126 |
| **IL-13** |  |  |  |  |  |  |  |  |  |
| rs10995604 | A | G | -0.157 | 0.034 | 4.48E-06 | 20.966 | 0.013 | 0.024 | 0.592 |
| rs117795020 | A | G | -0.358 | 0.072 | 5.48E-07 | 25.042 | -0.043 | 0.050 | 0.389 |
| rs12199215 | T | C | 0.131 | 0.028 | 4.16E-06 | 21.232 | -0.011 | 0.020 | 0.586 |
| rs12623722 | A | G | -0.119 | 0.026 | 3.61E-06 | 21.392 | 0.003 | 0.018 | 0.866 |
| rs13206012 | A | G | -0.372 | 0.026 | 1.85E-45 | 199.848 | 0.016 | 0.018 | 0.377 |
| rs13209117 | A | G | 0.141 | 0.028 | 6.76E-07 | 24.600 | -0.027 | 0.020 | 0.169 |
| rs138854806 | A | G | -0.420 | 0.084 | 5.45E-07 | 25.093 | -0.040 | 0.051 | 0.428 |
| rs139083458 | T | C | 1.000 | 0.211 | 2.17E-06 | 22.426 | -0.047 | 0.144 | 0.745 |
| rs145023524 | A | G | 0.282 | 0.059 | 1.66E-06 | 22.907 | -0.053 | 0.043 | 0.225 |
| rs150836197 | T | C | 0.328 | 0.071 | 4.14E-06 | 21.190 | -0.061 | 0.043 | 0.152 |
| rs27949 | T | C | -0.114 | 0.025 | 4.83E-06 | 20.928 | 0.020 | 0.018 | 0.275 |
| rs28442067 | A | G | -0.138 | 0.029 | 1.41E-06 | 23.236 | 0.015 | 0.021 | 0.478 |
| rs7073807 | T | C | 0.162 | 0.035 | 4.77E-06 | 20.879 | 0.021 | 0.025 | 0.403 |
| rs75383097 | C | G | -0.537 | 0.116 | 3.7E-06 | 21.411 | 0.018 | 0.068 | 0.789 |
| rs76339001 | A | T | -0.438 | 0.089 | 7.92E-07 | 24.370 | 0.054 | 0.052 | 0.296 |
| rs76975337 | T | C | -0.121 | 0.027 | 4.92E-06 | 20.872 | 0.002 | 0.019 | 0.905 |
| rs7747448 | A | G | -0.139 | 0.028 | 5.59E-07 | 25.094 | 0.034 | 0.020 | 0.085 |
| rs7757246 | T | C | 0.215 | 0.042 | 3.67E-07 | 25.870 | 0.027 | 0.031 | 0.388 |
| rs77955971 | A | C | 0.441 | 0.087 | 3.76E-07 | 25.775 | -0.027 | 0.047 | 0.562 |
| rs9296421 | T | G | 0.181 | 0.035 | 1.84E-07 | 27.157 | -0.011 | 0.023 | 0.637 |
| **IL-16** |  |  |  |  |  |  |  |  |  |
| rs117217798 | T | C | -0.206 | 0.044 | 2.77E-06 | 21.992 | -0.002 | 0.031 | 0.943 |
| rs142332135 | A | G | -0.765 | 0.108 | 1.58E-12 | 49.908 | 0.052 | 0.066 | 0.427 |
| rs144691581 | A | G | 0.493 | 0.096 | 2.67E-07 | 26.457 | -0.070 | 0.058 | 0.234 |
| rs35834666 | T | C | -0.173 | 0.035 | 6.57E-07 | 24.671 | -0.016 | 0.024 | 0.498 |
| rs4778640 | A | G | 0.719 | 0.098 | 2.55E-13 | 53.454 | -0.122 | 0.069 | 0.078 |
| rs4976691 | C | G | 0.125 | 0.026 | 1.47E-06 | 23.249 | -0.010 | 0.018 | 0.597 |
| rs7097884 | T | C | -0.119 | 0.024 | 8.81E-07 | 24.089 | 0.016 | 0.017 | 0.366 |
| rs78042619 | A | G | 0.550 | 0.116 | 2.02E-06 | 22.546 | -0.068 | 0.074 | 0.360 |
| **IL-9** |  |  |  |  |  |  |  |  |  |
| rs117807175 | C | G | -0.523 | 0.111 | 2.33E-06 | 22.306 | -0.044 | 0.076 | 0.566 |
| rs1259728 | A | G | -0.238 | 0.051 | 2.6E-06 | 22.043 | -0.059 | 0.037 | 0.113 |
| rs3736858 | C | G | -0.135 | 0.029 | 3.37E-06 | 21.542 | -0.034 | 0.021 | 0.112 |
| rs41294750 | T | C | 0.344 | 0.074 | 2.92E-06 | 21.859 | 0.049 | 0.051 | 0.333 |
| rs4880409 | T | C | -0.355 | 0.072 | 6.95E-07 | 24.597 | -0.148 | 0.084 | 0.080 |
| rs73443903 | A | C | 0.216 | 0.046 | 2.57E-06 | 22.078 | -0.011 | 0.034 | 0.754 |
| **MIF** |  |  |  |  |  |  |  |  |  |
| rs1007888 | T | C | -0.128 | 0.025 | 1.92E-07 | 27.067 | -0.008 | 0.018 | 0.632 |
| rs113218956 | A | G | -0.879 | 0.188 | 2.82E-06 | 21.937 | -0.046 | 0.128 | 0.722 |
| rs11551183 | C | G | 0.367 | 0.080 | 4E-06 | 21.252 | 0.047 | 0.059 | 0.425 |
| rs12594190 | A | G | 0.132 | 0.027 | 6.85E-07 | 24.649 | 0.016 | 0.019 | 0.396 |
| rs141009259 | T | C | -0.619 | 0.129 | 1.44E-06 | 23.221 | -0.199 | 0.075 | 0.008 |
| rs2294689 | C | G | -0.134 | 0.029 | 3.04E-06 | 21.722 | -0.026 | 0.072 | 0.719 |
| rs35792361 | A | G | -0.259 | 0.053 | 9.00E-07 | 24.065 | 0.000 | 0.036 | 0.996 |
| rs35890933 | T | G | 0.168 | 0.037 | 4.46E-06 | 21.073 | 0.030 | 0.024 | 0.214 |
| rs3814097 | A | G | -0.116 | 0.025 | 3.55E-06 | 21.457 | -0.013 | 0.017 | 0.461 |
| rs78098071 | T | C | -0.458 | 0.092 | 5.51E-07 | 25.073 | 0.022 | 0.068 | 0.746 |
| **TRAIL** |  |  |  |  |  |  |  |  |  |
| rs10084050 | A | G | -0.110 | 0.023 | 1.66E-06 | 22.909 | 0.005 | 0.025 | 0.847 |
| rs10164260 | A | G | 0.100 | 0.021 | 1.94E-06 | 22.591 | 0.002 | 0.023 | 0.921 |
| rs11081739 | A | G | 0.140 | 0.020 | 4.62E-12 | 47.680 | -0.031 | 0.022 | 0.155 |
| rs113057689 | A | G | -0.263 | 0.049 | 7.97E-08 | 28.809 | 0.037 | 0.041 | 0.365 |
| rs11875481 | T | C | -0.097 | 0.021 | 4.6E-06 | 21.085 | 0.049 | 0.023 | 0.033 |
| rs13115587 | A | C | 0.101 | 0.022 | 3.24E-06 | 21.658 | -0.039 | 0.024 | 0.114 |
| rs139958028 | A | G | 0.180 | 0.040 | 4.99E-06 | 20.830 | 0.035 | 0.040 | 0.376 |
| rs141032096 | T | G | -0.213 | 0.046 | 2.99E-06 | 21.807 | 0.056 | 0.039 | 0.156 |
| rs146827832 | T | C | 0.134 | 0.029 | 4E-06 | 21.231 | -0.031 | 0.028 | 0.281 |
| rs17535790 | A | G | -0.113 | 0.022 | 2.64E-07 | 26.625 | -0.064 | 0.024 | 0.008 |
| rs183815186 | A | T | -0.350 | 0.060 | 6.34E-09 | 33.775 | -0.015 | 0.063 | 0.813 |
| rs558572 | T | C | 0.135 | 0.027 | 3.42E-07 | 25.984 | -0.005 | 0.029 | 0.864 |
| rs57396456 | T | C | -0.564 | 0.052 | 7.71E-28 | 119.483 | 0.198 | 0.061 | 0.001 |
| rs62093482 | T | C | 0.983 | 0.053 | 6.12E-77 | 345.005 | -0.048 | 0.059 | 0.413 |
| rs7233927 | A | G | 0.091 | 0.016 | 3.63E-08 | 30.444 | -0.041 | 0.018 | 0.022 |
| rs73039026 | A | C | -0.310 | 0.063 | 1.02E-06 | 23.871 | 0.042 | 0.061 | 0.486 |
| rs73408359 | T | C | 0.415 | 0.036 | 3.47E-30 | 130.141 | -0.066 | 0.040 | 0.097 |
| rs74488044 | A | G | 0.347 | 0.033 | 2.26E-25 | 108.096 | 0.021 | 0.036 | 0.549 |
| rs747324 | T | C | -0.083 | 0.018 | 3.34E-06 | 21.529 | 0.009 | 0.019 | 0.634 |
| rs74778900 | T | C | 0.579 | 0.053 | 9.90E-28 | 118.908 | 0.015 | 0.074 | 0.839 |
| rs75473890 | T | C | -0.135 | 0.028 | 1.49E-06 | 23.206 | 0.026 | 0.031 | 0.407 |
| rs75489499 | T | C | -0.201 | 0.035 | 7.52E-09 | 33.412 | -0.060 | 0.034 | 0.075 |
| rs75928541 | A | G | 0.278 | 0.059 | 2.44E-06 | 22.185 | -0.007 | 0.058 | 0.900 |
| rs7599203 | T | C | 0.092 | 0.020 | 4.33E-06 | 21.063 | -0.012 | 0.022 | 0.577 |
| rs79085506 | A | G | 0.696 | 0.074 | 3.68E-21 | 89.187 | -0.106 | 0.073 | 0.147 |
| ***GH*** |  |  |  |  |  |  |  |  |  |
| **VEGF** |  |  |  |  |  |  |  |  |  |
| rs10411345 | C | G | -0.104 | 0.022 | 1.73E-06 | 22.796 | -0.031 | 0.019 | 0.100 |
| rs10757514 | C | G | -0.102 | 0.022 | 4.17E-06 | 21.270 | -0.007 | 0.021 | 0.751 |
| rs10822118 | T | C | -0.080 | 0.017 | 2.21E-06 | 22.500 | 0.008 | 0.016 | 0.621 |
| rs10934631 | T | C | -0.113 | 0.024 | 3.61E-06 | 21.518 | 0.009 | 0.022 | 0.670 |
| rs10967183 | T | C | -0.089 | 0.017 | 1.66E-07 | 27.539 | 0.020 | 0.016 | 0.199 |
| rs111950052 | A | G | -0.176 | 0.038 | 4.06E-06 | 21.183 | 0.017 | 0.032 | 0.585 |
| rs114773511 | T | C | 0.219 | 0.044 | 6.97E-07 | 24.587 | 0.000 | 0.038 | 0.995 |
| rs12156533 | A | T | 0.092 | 0.019 | 8.77E-07 | 24.193 | -0.012 | 0.017 | 0.490 |
| rs12456390 | T | C | -0.082 | 0.018 | 4.88E-06 | 20.878 | -0.009 | 0.017 | 0.595 |
| rs13190738 | T | C | 0.111 | 0.023 | 1.57E-06 | 23.123 | -0.010 | 0.017 | 0.558 |
| rs1730969 | C | G | -0.781 | 0.170 | 4.11E-06 | 21.203 | 0.063 | 0.139 | 0.650 |
| rs3025020 | T | C | -0.124 | 0.025 | 9.80E-07 | 24.013 | 0.015 | 0.018 | 0.395 |
| rs4573079 | A | C | 0.152 | 0.026 | 2.90E-09 | 35.337 | 0.016 | 0.022 | 0.456 |
| rs56071907 | T | C | 0.126 | 0.027 | 3.01E-06 | 21.772 | -0.005 | 0.023 | 0.842 |
| rs58078557 | A | T | -0.117 | 0.024 | 1.07E-06 | 23.678 | -0.010 | 0.023 | 0.656 |
| rs60987108 | A | G | 0.181 | 0.039 | 3.37E-06 | 21.581 | -0.037 | 0.035 | 0.295 |
| rs6496613 | A | C | -0.236 | 0.052 | 4.7E-06 | 20.976 | -0.058 | 0.049 | 0.236 |
| rs73872715 | T | C | -0.608 | 0.130 | 2.86E-06 | 21.894 | 0.113 | 0.112 | 0.315 |
| rs748227 | T | C | 0.238 | 0.036 | 6.61E-11 | 42.632 | -0.008 | 0.035 | 0.820 |
| rs7739450 | A | G | -0.415 | 0.018 | 9.01E-118 | 531.410 | 0.024 | 0.016 | 0.138 |
| rs7754905 | A | G | -0.130 | 0.020 | 1.79E-10 | 40.786 | 0.011 | 0.019 | 0.556 |
| rs7757024 | T | C | 0.144 | 0.029 | 7.02E-07 | 24.582 | -0.050 | 0.027 | 0.062 |
| rs77961527 | A | G | 0.229 | 0.046 | 5.53E-07 | 25.081 | 0.076 | 0.037 | 0.040 |
| rs9369440 | T | C | -0.087 | 0.019 | 2.83E-06 | 21.973 | 0.009 | 0.017 | 0.608 |
| rs9381249 | T | C | -0.241 | 0.040 | 1.04E-09 | 37.150 | 0.035 | 0.039 | 0.361 |
| rs9472153 | A | G | 0.106 | 0.017 | 1.04E-09 | 37.102 | -0.026 | 0.016 | 0.106 |
| **MIF** |  |  |  |  |  |  |  |  |  |
| rs1007888 | T | C | -0.128 | 0.025 | 1.92E-07 | 27.067 | 0.007 | 0.016 | 0.666 |
| rs113218956 | A | G | -0.879 | 0.188 | 2.82E-06 | 21.937 | -0.296 | 0.120 | 0.014 |
| rs11551183 | C | G | 0.367 | 0.080 | 4E-06 | 21.252 | 0.099 | 0.054 | 0.070 |
| rs12594190 | A | G | 0.132 | 0.027 | 6.85E-07 | 24.649 | 0.018 | 0.017 | 0.302 |
| rs141009259 | T | C | -0.619 | 0.129 | 1.44E-06 | 23.221 | -0.210 | 0.069 | 0.002 |
| rs2294689 | C | G | -0.134 | 0.029 | 3.04E-06 | 21.722 | 0.034 | 0.067 | 0.608 |
| rs35792361 | A | G | -0.259 | 0.053 | 9.00E-07 | 24.065 | -0.014 | 0.034 | 0.674 |
| rs35890933 | T | G | 0.168 | 0.037 | 4.46E-06 | 21.073 | 0.027 | 0.022 | 0.227 |
| rs3814097 | A | G | -0.116 | 0.025 | 3.55E-06 | 21.457 | -0.005 | 0.016 | 0.754 |
| rs78098071 | T | C | -0.458 | 0.092 | 5.51E-07 | 25.073 | 0.050 | 0.062 | 0.425 |
| ***EH*** |  |  |  |  |  |  |  |  |  |
| **MIP1b** |  |  |  |  |  |  |  |  |  |
| rs111942332 | T | G | -0.471 | 0.057 | 1.52E-16 | 68.053 | -0.066 | 0.098 | 0.498 |
| rs112337896 | A | G | 0.289 | 0.063 | 4.4E-06 | 21.051 | -0.025 | 0.094 | 0.790 |
| rs114933663 | T | C | 0.318 | 0.031 | 3.71E-25 | 107.134 | 0.025 | 0.058 | 0.662 |
| rs116237296 | A | G | 0.528 | 0.112 | 2.15E-06 | 22.453 | -0.112 | 0.225 | 0.618 |
| rs117084209 | C | G | 0.245 | 0.043 | 1.14E-08 | 32.626 | -0.054 | 0.113 | 0.628 |
| rs117139712 | T | C | -0.267 | 0.050 | 9.38E-08 | 28.487 | -0.055 | 0.093 | 0.558 |
| rs11716293 | C | G | 0.099 | 0.019 | 1.69E-07 | 27.210 | -0.011 | 0.036 | 0.757 |
| rs117394484 | T | C | -0.427 | 0.079 | 5.73E-08 | 29.450 | -0.040 | 0.110 | 0.718 |
| rs117453826 | A | G | -0.591 | 0.059 | 1.53E-23 | 99.874 | 0.042 | 0.105 | 0.691 |
| rs117503347 | T | C | 0.304 | 0.062 | 9.60E-07 | 24.020 | -0.133 | 0.078 | 0.089 |
| rs117657747 | A | G | 0.209 | 0.045 | 4.01E-06 | 21.259 | -0.020 | 0.066 | 0.757 |
| rs12452320 | A | C | 0.214 | 0.032 | 2.09E-11 | 44.951 | -0.008 | 0.060 | 0.890 |
| rs12951603 | A | G | -0.113 | 0.023 | 4.67E-07 | 25.306 | 0.013 | 0.043 | 0.767 |
| rs141793738 | A | G | 0.182 | 0.039 | 3.15E-06 | 21.765 | -0.053 | 0.075 | 0.478 |
| rs1437220 | T | C | 0.144 | 0.032 | 4.92E-06 | 20.806 | -0.030 | 0.056 | 0.590 |
| rs145526037 | T | G | -0.186 | 0.041 | 4.47E-06 | 21.051 | 0.196 | 0.078 | 0.011 |
| rs146565944 | T | C | 0.286 | 0.056 | 2.90E-07 | 26.319 | 0.002 | 0.084 | 0.982 |
| rs148561432 | A | G | -0.269 | 0.041 | 3.75E-11 | 43.705 | 0.055 | 0.063 | 0.381 |
| rs1543292 | A | G | 0.263 | 0.034 | 7.34E-15 | 60.392 | 0.057 | 0.056 | 0.309 |
| rs17138331 | A | G | -0.143 | 0.030 | 1.13E-06 | 23.624 | 0.016 | 0.055 | 0.768 |
| rs1867288 | C | G | 0.203 | 0.022 | 2.67E-21 | 89.479 | -0.013 | 0.038 | 0.721 |
| rs2276857 | T | C | -0.128 | 0.026 | 5.7E-07 | 24.916 | 0.043 | 0.050 | 0.386 |
| rs2314809 | T | C | -0.074 | 0.016 | 2.9E-06 | 21.911 | -0.006 | 0.030 | 0.847 |
| rs2373048 | A | T | -0.116 | 0.022 | 6.93E-08 | 29.103 | -0.049 | 0.040 | 0.216 |
| rs2376263 | A | G | 0.105 | 0.019 | 1.79E-08 | 31.701 | -0.044 | 0.037 | 0.236 |
| rs2673059 | T | C | 0.092 | 0.019 | 1.68E-06 | 23.004 | -0.012 | 0.037 | 0.741 |
| rs281728 | A | C | -0.079 | 0.017 | 3.89E-06 | 21.338 | 0.020 | 0.033 | 0.537 |
| rs28393318 | A | G | -0.108 | 0.024 | 4.62E-06 | 20.960 | 0.020 | 0.047 | 0.671 |
| rs28856610 | T | C | -0.307 | 0.050 | 5.12E-10 | 38.556 | 0.109 | 0.083 | 0.192 |
| rs34437725 | T | C | -0.257 | 0.048 | 9.17E-08 | 28.563 | -0.042 | 0.091 | 0.640 |
| rs35933743 | T | G | -0.118 | 0.024 | 6.56E-07 | 24.701 | -0.017 | 0.042 | 0.686 |
| rs41290648 | A | G | 0.223 | 0.024 | 9.21E-21 | 87.116 | -0.021 | 0.046 | 0.644 |
| rs41341749 | A | G | -0.166 | 0.029 | 1.67E-08 | 31.719 | 0.015 | 0.057 | 0.796 |
| rs41502550 | T | C | 0.127 | 0.022 | 8.13E-09 | 33.107 | 0.045 | 0.043 | 0.292 |
| rs4299194 | T | C | 0.089 | 0.019 | 2.33E-06 | 22.369 | -0.021 | 0.037 | 0.569 |
| rs4795931 | A | G | -0.087 | 0.017 | 6.10E-07 | 24.994 | -0.048 | 0.033 | 0.137 |
| rs4796110 | A | G | 0.124 | 0.026 | 1.15E-06 | 23.608 | -0.007 | 0.044 | 0.873 |
| rs56083628 | T | C | -0.130 | 0.026 | 4.45E-07 | 25.463 | 0.142 | 0.048 | 0.003 |
| rs62079535 | A | G | 0.231 | 0.039 | 2.91E-09 | 35.224 | -0.094 | 0.073 | 0.198 |
| rs6908843 | A | G | 0.100 | 0.021 | 1.78E-06 | 22.751 | -0.041 | 0.040 | 0.305 |
| rs72791296 | T | C | 0.236 | 0.047 | 3.97E-07 | 25.729 | -0.094 | 0.084 | 0.262 |
| rs72799710 | T | C | -0.104 | 0.022 | 1.79E-06 | 22.831 | -0.027 | 0.042 | 0.520 |
| rs72820112 | T | C | 0.106 | 0.018 | 7.80E-09 | 33.480 | -0.066 | 0.035 | 0.059 |
| rs72820246 | T | G | -0.098 | 0.017 | 4.37E-09 | 34.639 | -0.031 | 0.031 | 0.314 |
| rs72825991 | A | G | -0.179 | 0.031 | 9.44E-09 | 32.907 | 0.008 | 0.062 | 0.900 |
| rs74979864 | A | T | -0.318 | 0.061 | 2.03E-07 | 26.972 | 0.124 | 0.118 | 0.296 |
| rs76582507 | A | G | 0.326 | 0.068 | 1.42E-06 | 23.236 | -0.117 | 0.153 | 0.446 |
| rs76842834 | T | C | -0.421 | 0.047 | 4.46E-19 | 79.762 | 0.078 | 0.072 | 0.278 |
| rs76863419 | T | G | -0.283 | 0.034 | 9.77E-17 | 69.005 | 0.129 | 0.053 | 0.015 |
| rs79088462 | T | C | -0.321 | 0.058 | 3.77E-08 | 30.280 | -0.028 | 0.082 | 0.732 |
| rs80322601 | T | C | -0.192 | 0.038 | 4.69E-07 | 25.390 | 0.028 | 0.064 | 0.658 |
| rs8081726 | T | C | -0.350 | 0.036 | 2.74E-22 | 94.445 | 0.000 | 0.067 | 0.996 |
| rs854222 | A | C | 0.081 | 0.018 | 4.95E-06 | 20.886 | -0.008 | 0.034 | 0.819 |
| rs873944 | T | C | 0.239 | 0.032 | 1.03E-13 | 55.170 | -0.053 | 0.060 | 0.377 |
| rs939408 | A | C | -0.101 | 0.016 | 3.01E-10 | 39.602 | 0.052 | 0.031 | 0.099 |
| rs951814 | A | G | 0.161 | 0.032 | 2.92E-07 | 26.247 | -0.091 | 0.059 | 0.122 |
| rs9838883 | T | C | 0.079 | 0.017 | 1.79E-06 | 22.643 | -0.054 | 0.032 | 0.094 |
| rs9911839 | T | G | -0.138 | 0.027 | 3.52E-07 | 26.004 | 0.064 | 0.051 | 0.209 |
| rs9914803 | T | C | 0.098 | 0.016 | 3.47E-10 | 39.455 | 0.002 | 0.030 | 0.960 |
| **IL-18** |  |  |  |  |  |  |  |  |  |
| rs10409850 | A | G | 0.179 | 0.035 | 2.44E-07 | 26.625 | 0.037 | 0.045 | 0.415 |
| rs11214140 | T | C | -0.143 | 0.030 | 1.92E-06 | 22.590 | -0.013 | 0.036 | 0.720 |
| rs117266781 | T | C | 0.705 | 0.144 | 9.18E-07 | 24.097 | 0.306 | 0.175 | 0.080 |
| rs117371668 | T | G | 0.371 | 0.080 | 3.36E-06 | 21.572 | -0.141 | 0.103 | 0.174 |
| rs139468359 | T | C | 0.510 | 0.109 | 2.74E-06 | 21.969 | 0.246 | 0.133 | 0.065 |
| rs139727649 | T | C | -0.356 | 0.075 | 2.15E-06 | 22.459 | -0.056 | 0.103 | 0.586 |
| rs141091241 | T | C | -0.392 | 0.072 | 5.21E-08 | 29.596 | 0.079 | 0.082 | 0.335 |
| rs150005227 | T | C | 0.406 | 0.089 | 4.51E-06 | 21.008 | -0.041 | 0.112 | 0.716 |
| rs1979967 | T | C | 0.140 | 0.029 | 8.72E-07 | 24.117 | 0.038 | 0.037 | 0.299 |
| rs4952239 | A | T | -0.116 | 0.024 | 1.81E-06 | 22.806 | -0.047 | 0.031 | 0.132 |
| rs58701153 | A | T | -0.127 | 0.024 | 1.81E-07 | 27.310 | -0.015 | 0.032 | 0.642 |
| rs62312914 | T | C | -0.127 | 0.025 | 4.22E-07 | 25.590 | -0.032 | 0.031 | 0.304 |
| rs7599125 | A | G | 0.111 | 0.024 | 3.53E-06 | 21.519 | 0.029 | 0.031 | 0.342 |
| rs77187209 | T | C | -0.486 | 0.104 | 3.08E-06 | 21.775 | -0.106 | 0.126 | 0.401 |
| rs78623212 | T | C | 0.832 | 0.168 | 6.82E-07 | 24.642 | 0.195 | 0.176 | 0.266 |
| rs78716465 | A | G | 0.317 | 0.068 | 2.98E-06 | 21.825 | 0.111 | 0.083 | 0.184 |
| **TRAIL** |  |  |  |  |  |  |  |  |  |
| rs10084050 | A | G | -0.110 | 0.023 | 1.66E-06 | 22.909 | 0.021 | 0.045 | 0.641 |
| rs10164260 | A | G | 0.100 | 0.021 | 1.94E-06 | 22.591 | 0.064 | 0.041 | 0.115 |
| rs11081739 | A | G | 0.140 | 0.020 | 4.62E-12 | 47.680 | 0.006 | 0.039 | 0.878 |
| rs113057689 | A | G | -0.263 | 0.049 | 7.97E-08 | 28.809 | -0.043 | 0.072 | 0.552 |
| rs11875481 | T | C | -0.097 | 0.021 | 4.6E-06 | 21.085 | -0.012 | 0.041 | 0.769 |
| rs13115587 | A | C | 0.101 | 0.022 | 3.24E-06 | 21.658 | 0.021 | 0.043 | 0.626 |
| rs139958028 | A | G | 0.180 | 0.040 | 4.99E-06 | 20.830 | -0.001 | 0.072 | 0.987 |
| rs141032096 | T | G | -0.213 | 0.046 | 2.99E-06 | 21.807 | -0.072 | 0.069 | 0.300 |
| rs146827832 | T | C | 0.134 | 0.029 | 4E-06 | 21.231 | -0.002 | 0.051 | 0.966 |
| rs17535790 | A | G | -0.113 | 0.022 | 2.64E-07 | 26.625 | -0.131 | 0.043 | 0.003 |
| rs183815186 | A | T | -0.350 | 0.060 | 6.34E-09 | 33.775 | -0.116 | 0.109 | 0.286 |
| rs558572 | T | C | 0.135 | 0.027 | 3.42E-07 | 25.984 | 0.009 | 0.053 | 0.859 |
| rs57396456 | T | C | -0.564 | 0.052 | 7.71E-28 | 119.483 | -0.038 | 0.101 | 0.708 |
| rs62093482 | T | C | 0.983 | 0.053 | 6.12E-77 | 345.005 | -0.009 | 0.105 | 0.932 |
| rs7233927 | A | G | 0.091 | 0.016 | 3.63E-08 | 30.444 | -0.030 | 0.032 | 0.355 |
| rs73039026 | A | C | -0.310 | 0.063 | 1.02E-06 | 23.871 | -0.121 | 0.107 | 0.256 |
| rs73408359 | T | C | 0.415 | 0.036 | 3.47E-30 | 130.141 | 0.056 | 0.071 | 0.432 |
| rs74488044 | A | G | 0.347 | 0.033 | 2.26E-25 | 108.096 | -0.001 | 0.064 | 0.983 |
| rs747324 | T | C | -0.083 | 0.018 | 3.34E-06 | 21.529 | 0.038 | 0.034 | 0.263 |
| rs74778900 | T | C | 0.579 | 0.053 | 9.90E-28 | 118.908 | 0.226 | 0.132 | 0.088 |
| rs75473890 | T | C | -0.135 | 0.028 | 1.49E-06 | 23.206 | -0.077 | 0.055 | 0.162 |
| rs75489499 | T | C | -0.201 | 0.035 | 7.52E-09 | 33.412 | 0.001 | 0.060 | 0.987 |
| rs75928541 | A | G | 0.278 | 0.059 | 2.44E-06 | 22.185 | -0.098 | 0.104 | 0.346 |
| rs7599203 | T | C | 0.092 | 0.020 | 4.33E-06 | 21.063 | -0.023 | 0.038 | 0.549 |
| rs79085506 | A | G | 0.696 | 0.074 | 3.68E-21 | 89.187 | 0.140 | 0.126 | 0.266 |
| ***PF*** |  |  |  |  |  |  |  |  |  |
| **MIP1b** |  |  |  |  |  |  |  |  |  |
| rs111942332 | T | G | -0.471 | 0.057 | 1.52E-16 | 68.053 | -0.026 | 0.046 | 0.568 |
| rs112337896 | A | G | 0.289 | 0.063 | 4.4E-06 | 21.051 | 0.001 | 0.045 | 0.978 |
| rs114933663 | T | C | 0.318 | 0.031 | 3.71E-25 | 107.134 | -0.021 | 0.028 | 0.463 |
| rs116237296 | A | G | 0.528 | 0.112 | 2.15E-06 | 22.453 | -0.033 | 0.102 | 0.744 |
| rs117084209 | C | G | 0.245 | 0.043 | 1.14E-08 | 32.626 | -0.002 | 0.054 | 0.964 |
| rs117139712 | T | C | -0.267 | 0.050 | 9.38E-08 | 28.487 | 0.007 | 0.045 | 0.868 |
| rs11716293 | C | G | 0.099 | 0.019 | 1.69E-07 | 27.210 | -0.019 | 0.017 | 0.252 |
| rs117394484 | T | C | -0.427 | 0.079 | 5.73E-08 | 29.450 | 0.033 | 0.054 | 0.538 |
| rs117453826 | A | G | -0.591 | 0.059 | 1.53E-23 | 99.874 | 0.047 | 0.049 | 0.330 |
| rs117503347 | T | C | 0.304 | 0.062 | 9.60E-07 | 24.020 | -0.022 | 0.037 | 0.560 |
| rs117657747 | A | G | 0.209 | 0.045 | 4.01E-06 | 21.259 | -0.042 | 0.031 | 0.183 |
| rs12452320 | A | C | 0.214 | 0.032 | 2.09E-11 | 44.951 | -0.030 | 0.028 | 0.285 |
| rs12951603 | A | G | -0.113 | 0.023 | 4.67E-07 | 25.306 | 0.027 | 0.020 | 0.174 |
| rs141793738 | A | G | 0.182 | 0.039 | 3.15E-06 | 21.765 | 0.007 | 0.036 | 0.847 |
| rs1437220 | T | C | 0.144 | 0.032 | 4.92E-06 | 20.806 | 0.014 | 0.027 | 0.594 |
| rs145526037 | T | G | -0.186 | 0.041 | 4.47E-06 | 21.051 | 0.013 | 0.037 | 0.737 |
| rs146565944 | T | C | 0.286 | 0.056 | 2.90E-07 | 26.319 | 0.011 | 0.040 | 0.787 |
| rs148561432 | A | G | -0.269 | 0.041 | 3.75E-11 | 43.705 | -0.004 | 0.030 | 0.887 |
| rs1543292 | A | G | 0.263 | 0.034 | 7.34E-15 | 60.392 | 0.023 | 0.027 | 0.394 |
| rs17138331 | A | G | -0.143 | 0.030 | 1.13E-06 | 23.624 | -0.007 | 0.026 | 0.796 |
| rs1867288 | C | G | 0.203 | 0.022 | 2.67E-21 | 89.479 | -0.015 | 0.018 | 0.398 |
| rs2276857 | T | C | -0.128 | 0.026 | 5.7E-07 | 24.916 | 0.024 | 0.024 | 0.315 |
| rs2314809 | T | C | -0.074 | 0.016 | 2.9E-06 | 21.911 | 0.013 | 0.014 | 0.370 |
| rs2373048 | A | T | -0.116 | 0.022 | 6.93E-08 | 29.103 | -0.025 | 0.019 | 0.173 |
| rs2376263 | A | G | 0.105 | 0.019 | 1.79E-08 | 31.701 | -0.017 | 0.017 | 0.324 |
| rs2673059 | T | C | 0.092 | 0.019 | 1.68E-06 | 23.004 | 0.016 | 0.017 | 0.351 |
| rs281728 | A | C | -0.079 | 0.017 | 3.89E-06 | 21.338 | 0.013 | 0.016 | 0.412 |
| rs28393318 | A | G | -0.108 | 0.024 | 4.62E-06 | 20.960 | 0.010 | 0.022 | 0.640 |
| rs28856610 | T | C | -0.307 | 0.050 | 5.12E-10 | 38.556 | 0.041 | 0.040 | 0.299 |
| rs34437725 | T | C | -0.257 | 0.048 | 9.17E-08 | 28.563 | 0.016 | 0.043 | 0.708 |
| rs35933743 | T | G | -0.118 | 0.024 | 6.56E-07 | 24.701 | -0.001 | 0.020 | 0.971 |
| rs41290648 | A | G | 0.223 | 0.024 | 9.21E-21 | 87.116 | 0.018 | 0.022 | 0.413 |
| rs41341749 | A | G | -0.166 | 0.029 | 1.67E-08 | 31.719 | 0.047 | 0.027 | 0.086 |
| rs41502550 | T | C | 0.127 | 0.022 | 8.13E-09 | 33.107 | -0.019 | 0.020 | 0.347 |
| rs4299194 | T | C | 0.089 | 0.019 | 2.33E-06 | 22.369 | 0.011 | 0.018 | 0.537 |
| rs4795931 | A | G | -0.087 | 0.017 | 6.10E-07 | 24.994 | -0.015 | 0.015 | 0.328 |
| rs4796110 | A | G | 0.124 | 0.026 | 1.15E-06 | 23.608 | -0.026 | 0.021 | 0.207 |
| rs56083628 | T | C | -0.130 | 0.026 | 4.45E-07 | 25.463 | -0.001 | 0.024 | 0.969 |
| rs62079535 | A | G | 0.231 | 0.039 | 2.91E-09 | 35.224 | 0.013 | 0.036 | 0.721 |
| rs6908843 | A | G | 0.100 | 0.021 | 1.78E-06 | 22.751 | -0.016 | 0.019 | 0.399 |
| rs72791296 | T | C | 0.236 | 0.047 | 3.97E-07 | 25.729 | -0.007 | 0.039 | 0.868 |
| rs72799710 | T | C | -0.104 | 0.022 | 1.79E-06 | 22.831 | 0.029 | 0.020 | 0.149 |
| rs72820112 | T | C | 0.106 | 0.018 | 7.80E-09 | 33.480 | -0.001 | 0.017 | 0.976 |
| rs72820246 | T | G | -0.098 | 0.017 | 4.37E-09 | 34.639 | 0.018 | 0.015 | 0.209 |
| rs72825991 | A | G | -0.179 | 0.031 | 9.44E-09 | 32.907 | -0.017 | 0.030 | 0.560 |
| rs74979864 | A | T | -0.318 | 0.061 | 2.03E-07 | 26.972 | 0.039 | 0.055 | 0.482 |
| rs76582507 | A | G | 0.326 | 0.068 | 1.42E-06 | 23.236 | -0.078 | 0.073 | 0.286 |
| rs76842834 | T | C | -0.421 | 0.047 | 4.46E-19 | 79.762 | 0.029 | 0.034 | 0.387 |
| rs76863419 | T | G | -0.283 | 0.034 | 9.77E-17 | 69.005 | 0.025 | 0.025 | 0.316 |
| rs79088462 | T | C | -0.321 | 0.058 | 3.77E-08 | 30.280 | 0.069 | 0.038 | 0.074 |
| rs80322601 | T | C | -0.192 | 0.038 | 4.69E-07 | 25.390 | -0.007 | 0.031 | 0.828 |
| rs8081726 | T | C | -0.350 | 0.036 | 2.74E-22 | 94.445 | -0.012 | 0.031 | 0.707 |
| rs854222 | A | C | 0.081 | 0.018 | 4.95E-06 | 20.886 | -0.017 | 0.016 | 0.297 |
| rs873944 | T | C | 0.239 | 0.032 | 1.03E-13 | 55.170 | -0.039 | 0.029 | 0.178 |
| rs939408 | A | C | -0.101 | 0.016 | 3.01E-10 | 39.602 | 0.010 | 0.015 | 0.515 |
| rs951814 | A | G | 0.161 | 0.032 | 2.92E-07 | 26.247 | 0.022 | 0.027 | 0.413 |
| rs9838883 | T | C | 0.079 | 0.017 | 1.79E-06 | 22.643 | -0.018 | 0.015 | 0.246 |
| rs9911839 | T | G | -0.138 | 0.027 | 3.52E-07 | 26.004 | 0.002 | 0.024 | 0.947 |
| rs9914803 | T | C | 0.098 | 0.016 | 3.47E-10 | 39.455 | -0.003 | 0.014 | 0.814 |
| **IL-13** |  |  |  |  |  |  |  |  |  |
| rs10995604 | A | G | -0.157 | 0.034 | 4.48E-06 | 20.966 | 0.017 | 0.020 | 0.403 |
| rs117795020 | A | G | -0.358 | 0.072 | 5.48E-07 | 25.042 | -0.001 | 0.042 | 0.983 |
| rs12199215 | T | C | 0.131 | 0.028 | 4.16E-06 | 21.232 | -0.019 | 0.017 | 0.260 |
| rs12623722 | A | G | -0.119 | 0.026 | 3.61E-06 | 21.392 | 0.001 | 0.016 | 0.952 |
| rs13206012 | A | G | -0.372 | 0.026 | 1.85E-45 | 199.848 | 0.011 | 0.015 | 0.467 |
| rs13209117 | A | G | 0.141 | 0.028 | 6.76E-07 | 24.600 | -0.017 | 0.017 | 0.301 |
| rs138854806 | A | G | -0.420 | 0.084 | 5.45E-07 | 25.093 | -0.044 | 0.043 | 0.306 |
| rs139083458 | T | C | 1.000 | 0.211 | 2.17E-06 | 22.426 | -0.073 | 0.121 | 0.549 |
| rs145023524 | A | G | 0.282 | 0.059 | 1.66E-06 | 22.907 | -0.042 | 0.037 | 0.250 |
| rs150836197 | T | C | 0.328 | 0.071 | 4.14E-06 | 21.190 | -0.029 | 0.036 | 0.414 |
| rs27949 | T | C | -0.114 | 0.025 | 4.83E-06 | 20.928 | 0.024 | 0.015 | 0.124 |
| rs28442067 | A | G | -0.138 | 0.029 | 1.41E-06 | 23.236 | -0.005 | 0.018 | 0.793 |
| rs7073807 | T | C | 0.162 | 0.035 | 4.77E-06 | 20.879 | 0.012 | 0.021 | 0.563 |
| rs75383097 | C | G | -0.537 | 0.116 | 3.7E-06 | 21.411 | -0.015 | 0.057 | 0.788 |
| rs76339001 | A | T | -0.438 | 0.089 | 7.92E-07 | 24.370 | 0.059 | 0.044 | 0.178 |
| rs76975337 | T | C | -0.121 | 0.027 | 4.92E-06 | 20.872 | 0.001 | 0.016 | 0.946 |
| rs7747448 | A | G | -0.139 | 0.028 | 5.59E-07 | 25.094 | 0.025 | 0.017 | 0.133 |
| rs7757246 | T | C | 0.215 | 0.042 | 3.67E-07 | 25.870 | -0.014 | 0.026 | 0.588 |
| rs77955971 | A | C | 0.441 | 0.087 | 3.76E-07 | 25.775 | -0.049 | 0.040 | 0.224 |
| rs9296421 | T | G | 0.181 | 0.035 | 1.84E-07 | 27.157 | -0.007 | 0.019 | 0.725 |
| **IL-16** |  |  |  |  |  |  |  |  |  |
| rs117217798 | T | C | -0.206 | 0.044 | 2.77E-06 | 21.992 | -0.011 | 0.026 | 0.668 |
| rs142332135 | A | G | -0.765 | 0.108 | 1.58E-12 | 49.908 | 0.055 | 0.056 | 0.324 |
| rs144691581 | A | G | 0.493 | 0.096 | 2.67E-07 | 26.457 | -0.082 | 0.050 | 0.097 |
| rs35834666 | T | C | -0.173 | 0.035 | 6.57E-07 | 24.671 | -0.025 | 0.020 | 0.223 |
| rs4778640 | A | G | 0.719 | 0.098 | 2.55E-13 | 53.454 | -0.100 | 0.058 | 0.088 |
| rs4976691 | C | G | 0.125 | 0.026 | 1.47E-06 | 23.249 | -0.019 | 0.015 | 0.221 |
| rs7097884 | T | C | -0.119 | 0.024 | 8.81E-07 | 24.089 | 0.004 | 0.015 | 0.789 |
| rs78042619 | A | G | 0.550 | 0.116 | 2.02E-06 | 22.546 | -0.052 | 0.063 | 0.408 |
| **IL-9** |  |  |  |  |  |  |  |  |  |
| rs117807175 | C | G | -0.523 | 0.111 | 2.33E-06 | 22.306 | -0.024 | 0.064 | 0.704 |
| rs1259728 | A | G | -0.238 | 0.051 | 2.6E-06 | 22.043 | -0.025 | 0.031 | 0.426 |
| rs3736858 | C | G | -0.135 | 0.029 | 3.37E-06 | 21.542 | -0.029 | 0.018 | 0.105 |
| rs41294750 | T | C | 0.344 | 0.074 | 2.92E-06 | 21.859 | 0.048 | 0.043 | 0.262 |
| rs4880409 | T | C | -0.355 | 0.072 | 6.95E-07 | 24.597 | -0.114 | 0.071 | 0.107 |
| rs73443903 | A | C | 0.216 | 0.046 | 2.57E-06 | 22.078 | -0.007 | 0.029 | 0.814 |

Abbreviations: SNP, Single nucleotide polymorphism; F,F-statistic; OR, Odds ratio ; Pval, P-value; PE, Pre-eclampsia or eclampsia; MIP1b, Macrophage inflammatory protein 1-beta; IL-13, Interleukin-13; IL-16, Interleukin-16; IL-9, Interleukin-9; MIF, Macrophage migration inhibitory factor; TRAIL, Tumor necrosis factor-related apoptosis-inducing ligand; GH, Gestational hypertension; VEGF, Vascular endothelial growth factor; EH, Pre-existing hypertension complicating pregnancy, childbirth and the puerperium; IL-18, Interleukin-18.

**Supplementary Table 4.** Heterogeneity and pleiotropy tests of the causal effects of HDP on inflammatory cytokines.

| **Outcome** | **Heterogeneity** | | **MR Eggar** | | **MR PRESSO** |
| --- | --- | --- | --- | --- | --- |
|  | **Cochran’s Q** | **Rucker’s Q** | **intercept** | **p-intercept** | **Global test p** |
| **PE** |  |  |  |  |  |
| MCSF | 0.7909 | 0.7613 | 0.0083 | 0.6401 | 0.820 |
| VEGF | 0.9534 | 0.9384 | -0.0003 | 0.9755 | 0.954 |
| IL-12p70 | 0.4822 | 0.4319 | -0.0003 | 0.9726 | 0.508 |
| IL-1b | 0.4770 | 0.6505 | -0.0320 | 0.0450 | 0.519 |
| IL-1ra | 0.1394 | 0.1434 | -0.0166 | 0.3192 | 0.167 |
| IL-4 | 0.3430 | 0.2985 | -0.0007 | 0.9473 | 0.374 |
| IL-7 | 0.8727 | 0.8448 | -0.0035 | 0.8151 | 0.875 |
| IL-8 | 0.3073 | 0.3452 | -0.0206 | 0.1898 | 0.325 |
| **GH** |  |  |  |  |  |
| MIP1a | 0.4625 | 0.4216 | 0.0049 | 0.7746 | 0.466 |
| HGF | 0.7345 | 0.6980 | -0.0029 | 0.7975 | 0.760 |
| IL-18 | 0.1984 | 0.1705 | 0.0044 | 0.8186 | 0.201 |
| IL-8 | 0.7361 | 0.7264 | -0.0140 | 0.4096 | 0.749 |

Abbreviations: PE, Pre-eclampsia or eclampsia; MCSF, Macrophage colony stimulating factor; VEGF, Vascular endothelial growth factor; IL-12p70, Interleukin-12p70; IL-1b, Interleukin-1 beta; IL-1ra, Interleukin-1 receptor antagonist; IL-4, Interleukin-4; IL-7, Interleukin-7; IL-8, Interleukin-8; GH, Gestational hypertension; MIP1a, Macrophage inflammatory protein 1-alpha; HGF, Hepatocyte growth factor; IL-18, Interleukin-18.

**Supplementary Table 5.** Details of hypertensive disease of pregnancy predicting SNPs with inflammatory cytokines.

| **SNP** | **Effect allele** | **Other allele** | **Eaf** | **Log(OR)** | **Se** | **Pval** |
| --- | --- | --- | --- | --- | --- | --- |
| **PE** |  |  |  |  |  |  |
| rs10882397 | A | C | 0.598 | 0.083 | 0.017 | 1.93E-06 |
| rs10946495 | C | T | 0.396 | 0.082 | 0.017 | 1.85E-06 |
| rs115694633 | C | T | 0.016 | 0.291 | 0.063 | 3.75E-06 |
| rs116420012 | T | C | 0.013 | -0.421 | 0.085 | 7.34E-07 |
| rs11650519 | C | T | 0.336 | 0.082 | 0.018 | 4.21E-06 |
| rs11683991 | C | T | 0.684 | 0.087 | 0.019 | 2.68E-06 |
| rs1173771 | G | A | 0.586 | 0.082 | 0.017 | 2.23E-06 |
| rs11751616 | G | A | 0.048 | -0.198 | 0.042 | 2.61E-06 |
| rs142808027 | T | C | 0.022 | -0.306 | 0.064 | 1.88E-06 |
| rs148462858 | C | A | 0.034 | 0.213 | 0.045 | 1.92E-06 |
| rs149197698 | C | T | 0.020 | -0.313 | 0.066 | 1.87E-06 |
| rs167479 | G | T | 0.574 | 0.101 | 0.017 | 4.47E-09 |
| rs17030668 | G | A | 0.134 | 0.111 | 0.024 | 4.70E-06 |
| rs17037388 | G | A | 0.148 | -0.132 | 0.025 | 9.30E-08 |
| rs17116514 | G | C | 0.969 | -0.217 | 0.046 | 2.03E-06 |
| rs1902859 | C | T | 0.420 | 0.084 | 0.017 | 1.07E-06 |
| rs3219489 | G | C | 0.226 | -0.099 | 0.021 | 1.66E-06 |
| rs35997090 | T | C | 0.034 | 0.202 | 0.044 | 4.16E-06 |
| rs4245909 | G | A | 0.501 | -0.079 | 0.017 | 4.01E-06 |
| rs4774622 | T | A | 0.858 | 0.119 | 0.025 | 2.40E-06 |
| rs62205368 | A | G | 0.117 | 0.119 | 0.026 | 4.32E-06 |
| rs6585195 | C | G | 0.119 | 0.118 | 0.026 | 4.02E-06 |
| rs6683290 | G | T | 0.410 | -0.080 | 0.017 | 4.45E-06 |
| rs7027115 | G | A | 0.367 | 0.082 | 0.018 | 3.09E-06 |
| rs71539681 | T | C | 0.022 | 0.260 | 0.056 | 2.76E-06 |
| rs7313132 | C | T | 0.846 | -0.106 | 0.023 | 4.53E-06 |
| rs7318880 | T | C | 0.465 | 0.079 | 0.017 | 3.80E-06 |
| rs7350699 | T | G | 0.173 | 0.103 | 0.022 | 2.72E-06 |
| rs76159275 | A | G | 0.014 | 0.310 | 0.066 | 2.86E-06 |
| rs7650076 | C | T | 0.631 | -0.082 | 0.017 | 2.45E-06 |
| rs77284716 | C | T | 0.032 | 0.210 | 0.046 | 4.48E-06 |
| rs79252936 | A | G | 0.090 | -0.148 | 0.031 | 1.78E-06 |
| rs8111930 | G | A | 0.917 | -0.144 | 0.030 | 1.27E-06 |
| **GH** |  |  |  |  |  |  |
| rs1032778 | C | A | 0.338 | -0.079 | 0.017 | 2.52E-06 |
| rs10742277 | G | C | 0.336 | 0.076 | 0.017 | 4.67E-06 |
| rs10765970 | G | T | 0.153 | 0.111 | 0.021 | 1.87E-07 |
| rs10774624 | A | G | 0.598 | -0.074 | 0.016 | 4.64E-06 |
| rs11187789 | A | G | 0.240 | -0.096 | 0.019 | 3.16E-07 |
| rs112095618 | G | A | 0.005 | 0.453 | 0.096 | 2.63E-06 |
| rs116337444 | A | G | 0.104 | 0.118 | 0.025 | 2.24E-06 |
| rs1173743 | T | G | 0.543 | 0.083 | 0.016 | 1.54E-07 |
| rs118166269 | G | C | 0.006 | 0.444 | 0.095 | 3.19E-06 |
| rs1252103 | C | T | 0.297 | 0.083 | 0.017 | 1.30E-06 |
| rs138631764 | G | A | 0.004 | 0.555 | 0.111 | 6.07E-07 |
| rs1418883 | T | A | 0.784 | -0.086 | 0.019 | 4.81E-06 |
| rs145921017 | A | G | 0.132 | 0.110 | 0.023 | 2.14E-06 |
| rs167479 | G | T | 0.574 | 0.086 | 0.016 | 8.59E-08 |
| rs186696265 | T | C | 0.017 | 0.273 | 0.058 | 2.39E-06 |
| rs1893396 | T | G | 0.454 | -0.076 | 0.016 | 1.60E-06 |
| rs1902859 | C | T | 0.420 | 0.079 | 0.016 | 6.53E-07 |
| rs1976935 | T | C | 0.351 | -0.086 | 0.017 | 2.13E-07 |
| rs1979471 | G | C | 0.122 | -0.118 | 0.025 | 2.73E-06 |
| rs2278233 | C | T | 0.185 | -0.096 | 0.021 | 3.24E-06 |
| rs2333642 | A | G | 0.589 | -0.074 | 0.016 | 4.31E-06 |
| rs2704368 | G | A | 0.837 | -0.102 | 0.021 | 9.80E-07 |
| rs2966117 | T | G | 0.508 | 0.074 | 0.016 | 3.00E-06 |
| rs33999366 | G | A | 0.256 | -0.097 | 0.018 | 1.27E-07 |
| rs4538604 | A | G | 0.512 | 0.072 | 0.016 | 4.96E-06 |
| rs4794029 | C | T | 0.619 | -0.078 | 0.016 | 1.40E-06 |
| rs55724990 | A | G | 0.041 | -0.204 | 0.042 | 1.26E-06 |
| rs56360506 | T | G | 0.039 | 0.186 | 0.038 | 1.32E-06 |
| rs60028924 | G | A | 0.031 | -0.239 | 0.050 | 1.82E-06 |
| rs62205368 | A | G | 0.117 | 0.113 | 0.024 | 2.56E-06 |
| rs6544654 | C | T | 0.489 | -0.080 | 0.016 | 4.15E-07 |
| rs6797400 | G | A | 0.568 | 0.077 | 0.016 | 1.59E-06 |
| rs6823900 | A | G | 0.347 | -0.076 | 0.017 | 4.69E-06 |
| rs72837273 | T | C | 0.105 | -0.145 | 0.026 | 3.99E-08 |
| rs73042491 | A | G | 0.105 | -0.121 | 0.027 | 4.95E-06 |
| rs73789426 | G | C | 0.154 | -0.114 | 0.023 | 4.42E-07 |
| rs75649452 | C | T | 0.085 | 0.127 | 0.027 | 3.72E-06 |
| rs7901551 | G | A | 0.593 | 0.082 | 0.016 | 4.68E-07 |
| rs79714204 | T | C | 0.139 | -0.111 | 0.023 | 1.82E-06 |
| rs9429767 | A | G | 0.231 | -0.089 | 0.019 | 2.88E-06 |
| rs9809990 | G | A | 0.426 | -0.076 | 0.016 | 2.34E-06 |
| rs9879130 | G | A | 0.760 | 0.088 | 0.019 | 2.28E-06 |

Abbreviations: SNP, Single nucleotide polymorphism; Eaf, Effect attributable to the factor; OR, Odds ratio; Pval, P-value; PE, Pre-eclampsia or eclampsia; GH, Gestational hypertension; EH,Pre-existing hypertension complicating pregnancy, childbirth and the puerperium; PF, Pre-eclampsia or poor fetal growth.
